# FOXO1-regulated lncRNA CYP1B1-AS1 suppresses breast cancer cell proliferation by inhibiting neddylation

**DOI:** 10.1007/s10549-023-07090-z

**Published:** 2023-08-28

**Authors:** Li Tang, Da Wei, Xinyu Xu, Dongping Mo, Daofu Cheng, Feng Yan

**Affiliations:** 1 Department of Clinical Laboratory, Jiangsu Cancer Hospital and Jiangsu Institute of Cancer Research and the Affiliated Cancer Hospital of Nanjing Medical University, No. 42 Baiziting Road, Nanjing, 210009 People’s Republic of China; 2 Department of Surgery, Jiangsu Cancer Hospital and Jiangsu Institute of Cancer Research and the Affiliated Cancer Hospital of Nanjing Medical University, Nanjing, 210009 People’s Republic of China; 3 Department of Pathology, Jiangsu Cancer Hospital and Jiangsu Institute of Cancer Research and the Affiliated Cancer Hospital of Nanjing Medical University, Nanjing, 210009 People’s Republic of China

**Keywords:** Breast cancer, Long noncoding RNA, FOXO1, NAE1, NEDD8, Neddylation

## Abstract

**Purpose:**

Overactivated neddylation is considered to be a common event in cancer. Long non-coding RNAs (lncRNAs) can regulate cancer development by mediating post-translational modifications. However, the role of lncRNA in neddylation modification remains unclear.

**Methods:**

LncRNA cytochrome P450 family 1 subfamily B member 1 antisense RNA 1 (CYP1B1-AS1) expression in breast cancer tissues was evaluated by RT-PCR and TCGA BRCA data. Gain and loss of function experiments were performed to explore the role of CYP1B1-AS1 in breast cancer cell proliferation and apoptosis in vitro and in vivo. Luciferase assay, CHIP-qPCR assay, transcriptome sequencing, RNA-pulldown assay, mass spectrometry, RIP-PCR and Western blot were used to investigate the regulatory factors of CYP1B1-AS1 expression and the molecular mechanism of CYP1B1-AS1 involved in neddylation modification.

**Results:**

We found that CYP1B1-AS1 was down-regulated in breast cancer tissues and correlated with prognosis. In vivo and in vitro functional experiments confirmed that CYP1B1-AS1 inhibited cell proliferation and induced apoptosis. Mechanistically, CYP1B1-AS1 was regulated by the transcription factor, forkhead box O1 (FOXO1), and could be upregulated by inhibiting the PI3K/FOXO1 pathway. Moreover, CYP1B1-AS1 bound directly to NEDD8 activating enzyme E1 subunit 1 (NAE1) to regulate protein neddylation.

**Conclusion:**

This study reports for the first time that CYP1B1-AS1 inhibits protein neddylation to affect breast cancer cell proliferation, which provides a new strategy for the treatment of breast cancer by lncRNA targeting neddylation modification.

**Supplementary Information:**

The online version contains supplementary material available at 10.1007/s10549-023-07090-z.

## Introduction

Breast cancer is the most commonly diagnosed malignancy in women and the leading cause of cancer-related death [1]. Long noncoding RNAs (lncRNAs), which are functional RNAs with limited or no protein-coding capacity, can participate in the precise regulation of disease development by interacting with target molecules, and are considered to be one of the driving factors of tumorigenesis [2, 3].

Neddylation refers to the process by which the ubiquitin-like protein, neural precursor cell expressed developmentally down-regulated protein 8 (NEDD8), covalently and reversibly binds to substrate proteins [4]. As a broad post-translational modification (PTM), neddylation can regulate a variety of cellular processes, including cell cycle progression and differentiation, apoptosis, and proteolysis [5]. Neddylation conjugation cascade is achieved by a series of activities catalyzed by E1 activating-enzymes, E2 conjugating-enzymes, and E3 ligases [6]. Among them, NEDD8 activating enzyme (NAE), the only E1 activating enzyme identified so far, is a heterodimer formed by NEDD8 activating enzyme E1 subunit 1 (NAE1) and ubiquitin-like modification activating enzyme 3 (UBA3) [7]. Targeting NAE1 to destroy NEDD8-mediated protein transformation has become an attractive strategy to manage cancers [8, 9]. MLN4924, a potent inhibitor of NAE1, is being evaluated in multiple clinical trials as a novel cancer treatment strategy targeting neddylation [10].

Although increasing number of studies have emphasized the extensive involvement of lncRNAs in PTM regulation [11], their role in neddylation remains unclear. In this study, we report for the first time that lncRNA cytochrome P450 family 1 subfamily B member 1 antisense RNA 1 (CYP1B1-AS1) can inhibit breast cancer cell proliferation and promote apoptosis. CYP1B1-AS1 is regulated by the transcription factor forkhead box O1 (FOXO1) and can inhibit the protein neddylation by binding to NAE1. Our study reveals the unknown biological function of lncRNA CYP1B1-AS1 and suggests a novel mechanism by which lncRNA regulates tumor pathogenesis through neddylation.

## Material and methods

### Clinical tissue specimens and breast cancer cell lines

Breast cancer and adjacent normal tissues of 44 female patients undergoing breast cancer surgery in 2021 were collected through the Biobank of Jiangsu Cancer Hospital. At least two pathologists performed histopathological diagnoses on the samples. Breast cancer cell lines MCF7, T-47D, SK-BR-3, BT549, MDA-MB-231, and breast cancer epithelial cell line MCF10A were obtained from the Cell Bank of the Chinese Academy of Sciences (Shanghai, China), cultured according to ATCC recommended procedures.

### RNA extraction and quantitative real-time polymerase chain reaction (qPCR)

Total RNA was isolated from cells and tissues using TRIzol reagent (Invitrogen, Carlsbad, CA, USA). The PARIS kit (Invitrogen) was used to isolate RNA in the nucleus and cytoplasm. cDNA preparation was carried out exactly according to the instructions of PrimeScript RT reagent Kit with gDNA Eraser (Takara, Tokyo, Japan). qPCR amplification was performed according to the TB Green Premix Ex Taq II (Takara) system. The relative expression levels of target genes were calculated by the 2^−ΔΔCt^ method, and the primer sequences were shown in Table S1.

### Fluorescence in situ hybridization (FISH)

CYP1B1-AS1 probe was synthesized and labeled with Cy3, and the probe sequence was shown in Table S2. The experimental procedure is consistent with our previous study [12].

### Small interfering RNAs (siRNAs), overexpression vectors and inhibitors

The sequences of siRNAs and negative control are shown in Table S3. Lentiviral vectors have been widely used in animal models and clinical studies, and their expression is considered effective and safe [13]. Lentiviral overexpression particles of CYP1B1-AS1 were synthesized and packaged by GenePharma (Shanghai, China) (Data S1). MCF7 and MDA-MB-231 cells were infected at multiplicity of infection (MOI) = 15. After 72 h, puromycin (5 μg/mL) was added to screen the cell lines stably overexpressing CYP1B1-AS1 and their negative controls, which were labeled as MCF7-exp and MCF7-NC, MDA-231-exp and MDA-231-NC, respectively. FOXO1 was cloned into the pcDNA3.1 vector and labeled p-FOXO1 (Data S2). Both siRNAs and plasmid DNA were transfected with Lipofectamine 3000 (Invitrogen), and all operations were performed according to the instructions. 5-azacytidine (5 μM, MedChemExpress, Monmouth Junction, NJ, USA) was used to treat MCF7 and MDA-MB-231 cells with medium changes every 24 h for 48 h. LY294002 (25 μM, MedChemExpress) was used to treat MCF7 and MDA-MB-231 cells for 48 h.

### Chromatin immunoprecipitation (ChIP)

The cells were seeded in a 10 cm diameter petri dish, and when the confluence reached 70–90%, a final concentration of 1% formaldehyde was added for cross-linking. After terminating the cross-linking with glycine, the cells were collected and added to a cell lysate containing protease inhibitors for cryogenic sonication. All procedures were performed in strict accordance with the instructions of the EZ-ChIP Chromatin Immunoprecipitation Kit (Sigma-Aldrich, St. Louis, MO, USA). qPCR was performed on the DNA obtained by co-precipitation, and the primer sequences are shown in Table S4.

### Xenograft model

MCF7-exp cells stably upregulated expressing CYP1B1-AS1 and their negative controls were resuspended in 0.1 mL PBS (> 2 × 10^6^ cells) and injected into the fat pads of 5–6 weeks old female BALB/c nude mice. The tumor growth curves were plotted in a manner consistent with our previous study [12]. After 31 days, the mice were sacrificed, and the tumor tissues were taken out and weighed. Tissue paraffin sections were prepared for subsequent detection.

### Cell proliferation assay

Cell proliferation ability was detected using Cell Counting Kit-8 (CCK-8) (Dojindo, Kumamoto, Japan). The cells were seeded in a 96-well culture plate (3 × 10^3^ cells/well). The following experimental procedure is consistent with our previous study [12].

### RNA pull-down and high-performance liquid chromatography–mass spectrometry (LC–MS/MS)

Experiments were performed using the Pierce Magnetic RNA–Protein Pull-Down Kit (Thermo Fisher Scientific, Waltham, MA, USA). The pull-down protein solutions were detected with LC–MS/MS system consisting of an L-3000 high performance liquid chromatography system (RIGOL, Beijing, China) and an Orbitrap Exploris 480 mass spectrometer (Thermo Fisher Scientific), and the results were analyzed with Proteome Discoverer 2.4 software. The primers are listed in Table S5.

### Western blot

The experimental procedure is consistent with our previous study [14]. The dilutions of specific antibodies were: anti-FOXO1 (1:1000), NAE1 (1:1000), B-cell lymphoma 2 apoptosis regulator (BCL2, 1:1000), B-cell lymphoma 2 associated X (BAX, 1:1000), cyclin D1 (1:1000), p21 (1:1000), NEDD8 (1:1000), UAB3 (1:1000), and beta-actin (1:1000). Beta-Actin was used as an internal reference. Antibodies are listed in Table S6.

### Statistical analysis

The Wilcoxon matched-pairs signed-rank test was used to compare matched breast cancer and paracancerous tissue samples. Student's *T* test and analysis of variance were used for comparison between groups. All data are representative of at least three independent experiments. *P* values less than 0.05 were considered statistically significant. Data were analyzed using GraphPad Prism 8 (GraphPad Software, San Diego, CA, USA).

## Results

### CYP1B1-AS1 downregulation is associated with breast cancer progression

**Fig. 1:**
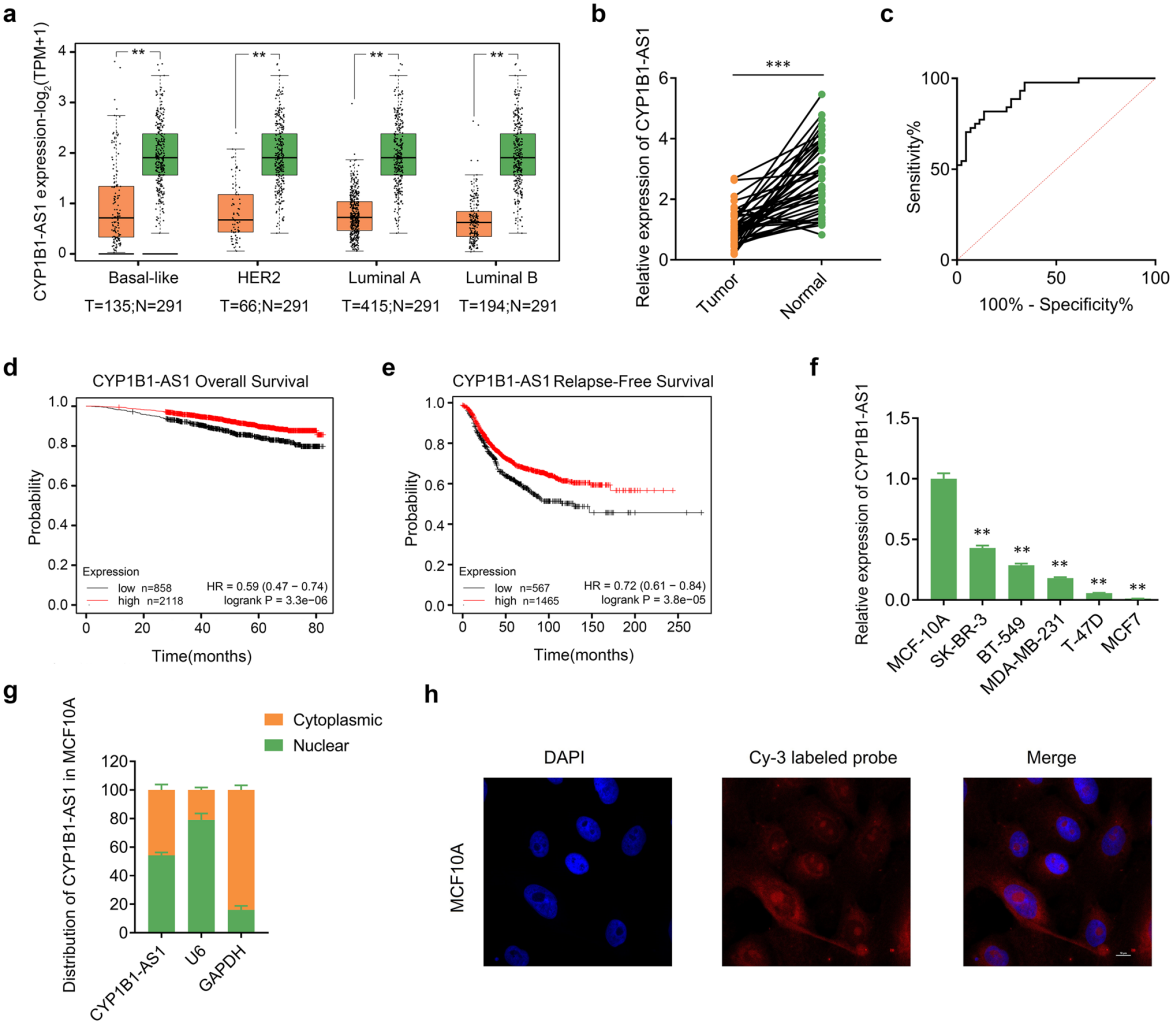
Downregulation of CYP1B1-AS1 is associated with breast cancer progression. **a** The Cancer Genome Atlas (TCGA) data analysis of CYP1B1-AS1 expression in each molecular subtype of breast cancer. N, non-tumor tissue. T, tumor tissue. **P < 0.01. **b** Quantitative real-time polymerase chain reaction (qPCR) was used to analyze the expression level of CYP1B1-AS1 in breast cancer tissues and adjacent non-tumor tissues of 44 patients. ***P < 0.001. **c** Receiver operating characteristic curve analysis of the diagnostic value of CYP1B1-AS1 in 44 paired breast tissue samples. *P* < 0.001. **d** Kaplan–Meier Plotter analysis of the relationship between CYP1B1-AS1 expression and patients’ overall survival. Kaplan–Meier Plotter uses data from public databases such as GEO and TCGA and provides the best available cut-off value. The cut-off value used in the analysis is 4. *P* < 0.001. **e** Kaplan–Meier Plotter analysis of the relationship between CYP1B1-AS1 expression and patients’ recurrence-free survival. Kaplan–Meier Plotter uses data from public databases such as GEO and TCGA and provides the best available cut-off value. The cut-off value used in the analysis is 1.28. *P* < 0.001. **f** qPCR was used to analyze the expression level of CYP1B1-AS1 in breast cancer cells (BT-549, SK-BR-3, MDA-MB-231, T-47D, and MCF7), and the mammary epithelial cell, MCF10A, was used as control. **P < 0.01. **g** Nuclear/cytoplasmic separation combined with qPCR were used to analyze the distribution of CYP1B1-AS1 in MCF10A cells. U6 and GAPDH were used as the reference for nuclear RNA extraction and cytoplasmic RNA extraction, respectively. **h** Fluorescence in situ hybridization (FISH) experiments combined with laser confocal microscopy showed the distribution of CYP1B1-AS1 in MCF10A cells (× 1000)

In our previous work, based on the lncRNA microarray data (GSE115275), the expression of CYP1B1-AS1 was downregulated in breast cancer (fold change = 3.3522976, *P* < 0.05) [15]. *CYP1B1-AS1* is located on the sense strand of human chromosome 2 with a length of 1776 nt and contains a poly A tail (NR_027252.1), is in a tail-to-tail relationship with the host gene *CYP1B1*, and has no sequence in common. LNCipedia (https://lncipedia.org) showed that CYP1B1-AS1 is poorly conserved between species (PhyloCSF score = − 77.3697), and the coding probability was only 16.57% (Fig. S1a). The open reading frames (ORFs) finder (https://www.ncbi.nlm.nih.gov/orffinder/) indicated the presence of some short ORFs in the *CYP1B1-AS1* sequence, but BLAST (https://blast.ncbi.nlm.nih.gov/Blast.cgi) showed that there was no match between the predicted short peptide sequences and the known protein amino acid sequences. GEPIA2 (http://gepia2.cancer-pku.cn/#index) showed that CYP1B1-AS1 was downregulated in luminal A, luminal B, human epidermal growth factor receptor 2 (HER2) and basal-like breast cancers (Fig. 1a), but did not differ between these subtypes (Fig. S1b). Moreover, CYP1B1-AS1 downregulation was also seen in 10 other solid tumors (Fig. S1c). The detection results of 44 breast cancer clinical samples that we collected showed that CYP1B1-AS1 was significantly downregulated in cancer tissues compared with adjacent normal tissues (Fig. 1b). The receiver operating characteristic (ROC) curve analysis suggested that CYP1B1-AS1 has a high diagnostic value (AUC = 0.918, 95% confidence interval, 0.864–0.972, *P* < 0.001) (Fig. 1c). Kaplan–Meier Plotter (http://kmplot.com/analysis/index.php) showed that breast cancer patients with high CYP1B1-AS1 expression had better overall survival and recurrence-free survival (Fig. 1d, e). The detection results of breast cancer cell lines showed that the expression of CYP1B1-AS1 in MCF7, T47D, MDA-MB-231, SK-BR-3, and BT-549 cells was significantly lower than that in mammary epithelial cells, MCF10A (Fig. 1f). FISH experiments combined with RNA nuclear/cytoplasmic separation detection showed that CYP1B1-AS1 was distributed in both the nucleus and cytoplasm of MCF10A cells, with a distribution percentage of 54.2% and 45.8%, respectively (Fig. 1g, h).

### CYP1B1-AS1 downregulation is associated with transcription factor, FOXO1

**Fig. 2:**
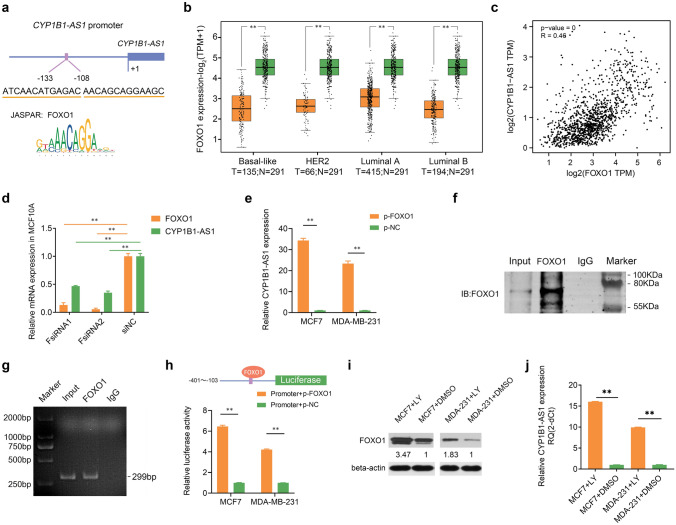
The upstream regulatory mechanism of CYP1B1-AS1. **a** Schematic representation of the binding sites of FOXO1 predicted by JASPAR in the CYP1B1-AS1 promoter region. **b** The Cancer Genome Atlas (TCGA) data were analyzed to determine the expression of FOXO1 in various molecular subtypes of breast cancer. N, non-tumor tissue. T, tumor tissue. **P < 0.01. **c** TCGA data were analyzed to determine the correlation between CYP1B1-AS1 and FOXO1 expression in breast cancer. *R*, Pearson correlation coefficient. *P* < 0.001. **d** Quantitative real-time polymerase chain reaction (qPCR) analysis of the effect of interfering FOXO1 on CYP1B1-AS1 expression in MCF10A cells. FsiRNA, FOXO1 siRNA. siNC, negative control for siRNA. **P < 0.01. **e** qPCR analysis of the effect of up-regulated FOXO1 expression on CYP1B1-AS1 expression in MCF7 and MDA-MB-231 cells. p-FOXO1, pcDNA3.1-FOXO1. p-NC, negative control for pcDNA3.1. **P < 0.01. **f** Western blot results of chromatin immunoprecipitation (CHIP) assay to detect FOXO1 binding to *CYP1B1-AS1* promoter fragment. Input, positive control. IgG, negative control. **g** Gel electropherogram of the amplified promoter fragment in CHIP-qPCR assay. Input, positive control. IgG, negative control. **h** Dual-luciferase assay was used to detect the downstream regulation of FOXO1 binding to the promoter fragment. p-FOXO1, pcDNA3.1-FOXO1. p-NC, negative control for pcDNA3.1. **P < 0.01. **i** Western blot was used to detect the changes in FOXO1 protein expression after LY294002 treatment. LY, LY294002. Solvent DMSO as blank control. **j** qPCR was used to detect changes in CYP1B1-AS1 expression after LY294002 treatment. LY, LY294002. Solvent DMSO as blank control. **P < 0.01

To explore the regulatory mechanism of CYP1B1-AS1 down-regulation in breast cancer, we first used the UCSC Genome Browser (http://genome.ucsc.edu) and EBI CpGplot (http://www.ebi.ac.uk/Tools/seqstats/emboss_cpgplot/) to analyze the *CYP1B1-AS1* promoter (2000 bp upstream), and no CpG islands, which are considered as the best predictors for defining active or potentially active promoter regions [16], were found (Fig. S2a, b). Furthermore, treatment of MCF7 and MDA-MB-231 cells with the demethylating drug 5-azacytidine also did not significantly alter the expression of CYP1B1-AS1 (Fig. S2c). JASPAR (https://jaspar.genereg.net) suggested that there are multiple binding sites for the tumor suppressor transcription factor, FOXO1, in the *CYP1B1-AS1* promoter region (Fig. 2a, Fig. S2d). The Cancer Genome Atlas (TCGA) (https://portal.gdc.cancer.gov) data showed that FOXO1 was downregulated in luminal A, luminal B, HER2, and basal-like breast cancers (Fig. 2b), and positively correlated with CYP1B1-AS1 expression (*R* = 0.46, Fig. 2c). By interfering with FOXO1 in MCF10A cells, CYP1B1-AS1 was downregulated (Fig. 2d). By overexpressing FOXO1 in MCF7 and MDA-MB-231 cells, CYP1B1-AS1 was upregulated (Fig. 2e). The fragment with the highest score and containing three consecutive binding sites was selected for CHIP assay, and the results showed that FOXO1 was able to bind to this promoter (Fig. 2f, g). Dual-luciferase experiments also indicated that FOXO1 could promote the expression of downstream luciferase proteins by binding to this fragment (Fig. 2h). Considering that the active PI3K signaling pathway in tumor cells destabilized FOXO1 protein [17], we treated MCF7 and MDA-MB-231 cells with the phosphatidylinositol-3-kinase (PI3K) inhibitor, LY294002, and found that the expressions of FOXO1 protein and CYP1B1-AS1 were up-regulated (Fig. 2i, j).

### CYP1B1-AS1 inhibits cell proliferation and induces apoptosis

**Fig. 3:**
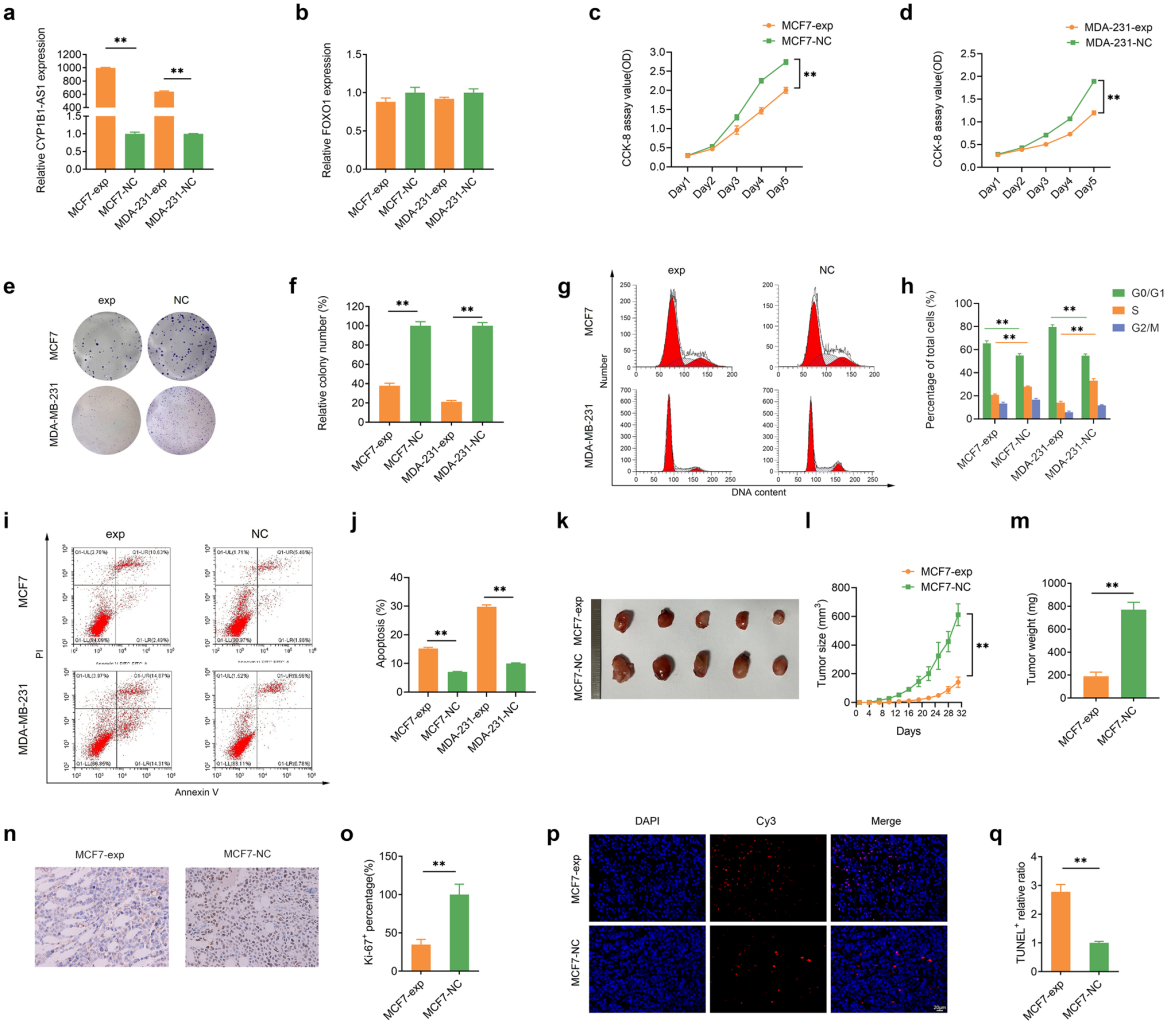
Upregulation of CYP1B1-AS1 inhibits cell proliferation and induces apoptosis. **a** Quantitative real-time polymerase chain reaction (qPCR) was used to detect the changes of CYP1B1-AS1 after lentivirus infection of MCF7 and MDA-MB-231 cells. **P < 0.01. **b** qPCR was used to detect FOXO1 mRNA in MCF7 and MDA-MB-231 cells after up-regulation of CYP1B1-AS1. **c**, **d** Counting Kit 8 (CCK-8) was used to detect the changes in the proliferation ability of MCF7 and MDA-MB-231 cells after up-regulation of CYP1B1-AS1. **P < 0.01. **e**, **f** The clone formation assay was used to examine the effect of up-regulation of CYP1B1-AS1 on the clone formation ability of breast cancer single cells. **P < 0.01. **g**, **h** Flow cytometry was used to analyze the effect of up-regulation of CYP1B1-AS1 on breast cancer cell cycle. **P < 0.01. **i**, **j** Flow cytometry was used to analyze the effect of up-regulation of CYP1B1-AS1 on apoptosis. **P < 0.01. **k, m** Representative images of tumor tissue in the xenograft model and compared the volume and weight of tumor tissue in the MCF7-exp and MCF7-NC groups (*n* = 6). Tumor volumes shown in **l** were calculated every 3 days after injection. **P < 0.01. **n**, **o** Representative images of immunohistochemical staining showing the expression levels of Ki-67 in MCF7-exp and MCF7-NC transplanted tumor tissues (× 400). **P < 0.01. **p**, **q** Representative images of TUNEL fluorescence staining showing the level of apoptosis in MCF7-exp and MCF7-NC transplanted tumor tissues (× 400). **P < 0.01

We used the recombinant lentivirus of CYP1B1-AS1 to infect MCF7 and MDA-MB-231 cells in functional experiments. qPCR results showed that CYP1B1-AS1 was up-regulated by about 997-fold in MCF7 cells (MCF7-exp vs. MCF7-NC), and CYP1B1-AS1 was upregulated by about 639-fold in MDA-MB-231 cells (MCF7-exp vs. MCF7-NC) (Fig. 3a). The results of RNA-Seq and qPCR showed no change in FOXO1 mRNA after upregulating CYP1B1-AS1 expression (Fig. 3b). CCK-8 assay showed that the up-regulation of CYP1B1-AS1 could significantly inhibit the proliferation of MCF7 and MDA-MB-231 cells (Fig. 3c, d). In addition, the number of clones formed by MCF7-exp and MDA-231-exp cells was reduced to 37.89% and 21.16% of the control groups, respectively (Fig. 3e, f). Cell cycle analysis showed that after up-regulation of CYP1B1-AS1, the percentage of G1 phase distribution of MCF7 cells increased from 55.05 to 65.62%, and the percentage of S phase distribution decreased from 28.15 to 20.94%. Similarly, the percentage of G1 phase distribution of MDA-MB-231 cells increased from 54.91 to 79.82%, and the percentage of S phase distribution decreased from 33.19 to 14.20% (Fig. 3g, h). Apoptosis analysis showed that the upregulation of CYP1B1-AS1 increased the percentage of apoptosis from 7.05 to 15.22% in MCF7 cells and from 9.97 to 29.78% in MDA-MB-231 cells (Fig. 3i, j). Xenograft model studies showed that tumor grafts formed by MCF7-exp cells were smaller in mass and volume compared to those of negative controls (Fig. 3k–m). Immunohistochemical staining showed that Ki-67 expression was significantly reduced in MCF7-exp tumor grafts (Fig. 3n, o). TUNEL staining showed that apoptotic cells were significantly increased in MCF7-exp tumor grafts (Fig. 3p, q).

### CYP1B1-AS1 is extensively involved in intracellular regulation

**Fig. 4:**
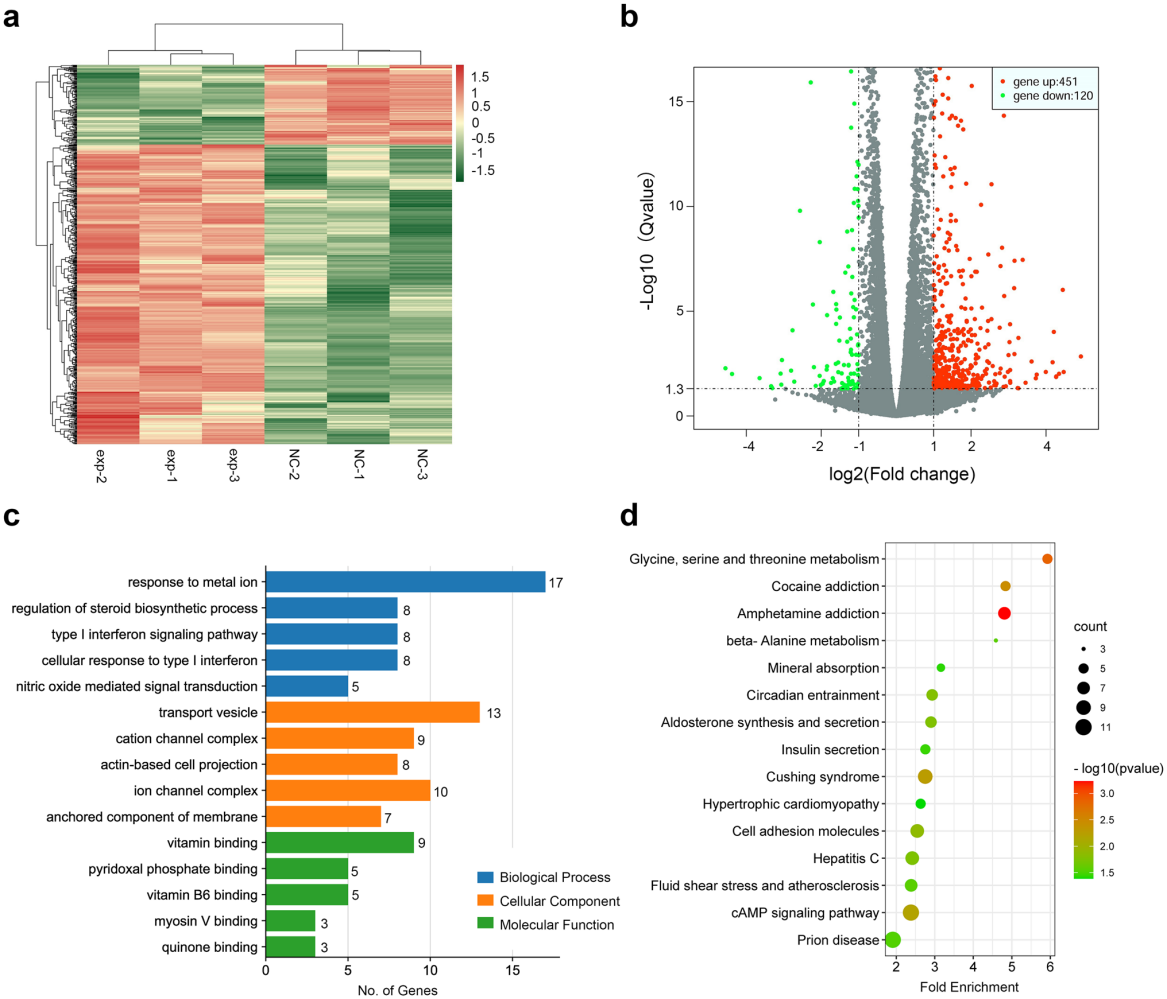
Transcriptome sequencing analysis of MCF7 cells following CYP1B1-AS1 upregulation. **a** Cluster heatmap of differential gene expression analysis between MCF7-exp and MCF7-NC groups. **b** Volcano plots show the distribution of differentially expressed genes between the MCF7-exp and MCF7-NC groups. Q, the corrected *P*-value. **c** Horizontal bar graph showing Gene Ontology enrichment analysis of differentially expressed genes, and the top five items with *P* < 0.05 are shown for each category. **d** Dot plot showing Kyoto Encyclopaedia of Genes and Genomes pathway enrichment analysis of differentially expressed genes, and the top 15 items with *P* < 0.05 are shown

To understand the global regulation of gene expression by CYP1B1-AS1 in breast cancer cells, RNA sequencing was performed. Using the fold difference (|log_2_(fold change) |> 1) and significant level (*Q* value < 0.05) as screening conditions, we found that a total of 571 genes were differentially expressed in MCF7 cells that up-regulated CYP1B1-AS1. Among them, 451 genes were upregulated and 120 genes were downregulated (Fig. 4a, b). Gene Ontology (GO) enrichment analysis showed that these genes were involved in 368 biological processes, had 69 molecular functions, and were distributed in 41 cellular components (Fig. 4c). Kyoto Encyclopedia of Genes and Genomes (KEGG) enrichment analysis showed that upregulation of CYP1B1-AS1 expression could affect multiple biological pathways (Fig. 4d).

### CYP1B1-AS1 binds directly to NAE1

**Fig. 5:**
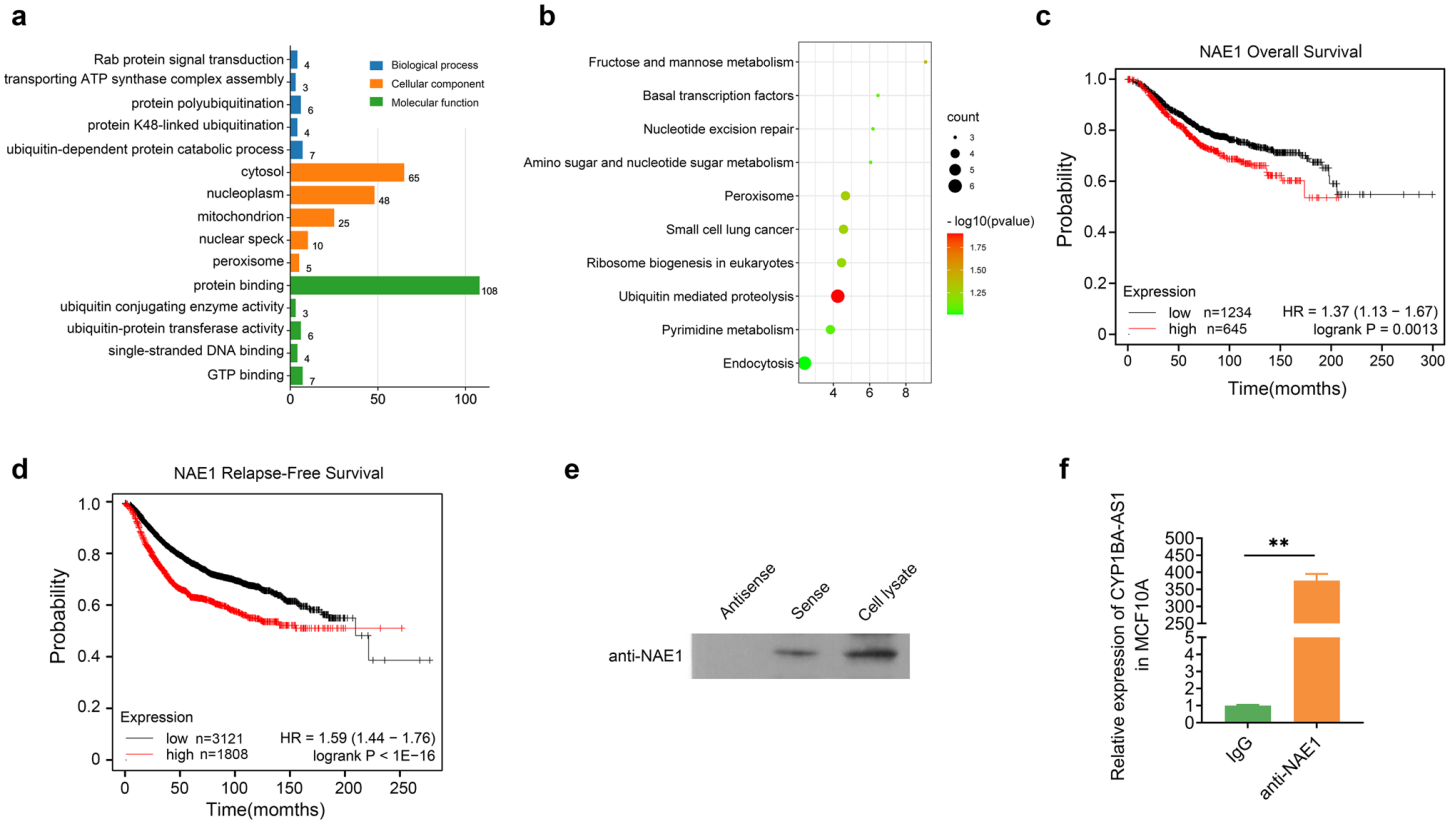
CYP1B1-AS1 binds directly to NAE1 protein. **a** Gene Ontology enrichment analysis of CYP1B1-AS1 pull-down proteins, each class showing the top five items of *P* < 0.05. **b** Kyoto Encyclopaedia of Genes and Genomes pathway enrichment analysis of CYP1B1-AS1 pull-down proteins, showing all items with *P* < 0.05. **c** Kaplan–Meier Plotter analysis of the relationship between NAE1 expression and overall survival in breast cancer patients. *P* < 0.01. **d** Kaplan–Meier Plotter analysis of the relationship between NAE1 expression and recurrence-free survival in breast cancer patients. *P* < 0.001. **e** Western blot was used to detect NAE1 protein in CYP1B1-AS1 sense strand pull-down protein solution. CYP1B1-AS1 antisense strand pull-down protein solution was used as a negative control, and MCF7-exp whole cell lysate was used as a positive control. **f** The RNA immunoprecipitation products were isolated and purified, and the amount of CYP1B1-AS1 bound to anti-NAE1 or IgG was measured by qPCR. IgG was used as a negative control. **P < 0.01

We performed RNA pull-down combined with LC–MS/MS detection to explore the functional pathway of CYP1B1-AS1, and finally obtained 134 proteins that specifically bind to the sense strand of CYP1B1-AS1. GO and KEGG enrichment analyses of these CYP1B1-AS1 binding proteins using the DAVID database (https://david.ncifcrf.gov) showed that these proteins are widely distributed in the nucleus and cytoplasm and are involved in important biological processes such as proteolysis (Fig. 5a, b). Survival analysis showed that breast cancer patients with high NAE1 expression had poorer overall survival and recurrence-free survival (Fig. 5c, d). Western blot analysis showed that NAE1 could only be detected in the pull-down protein solution of sense CYP1 B1-AS1 (Fig. 5e). We also performed RNA immunoprecipitation (RIP) experiments to precipitate NAE1 protein in MCF10A cell lysates, and detected that CYP1B1-AS1 was bound to it, confirming that CYP1B1-AS1 can stably bind to NAE1 protein (Fig. 5f).

### CYP1B1-AS1 regulates cell proliferation and apoptosis by affecting neddylation through NAE1

**Fig. 6:**
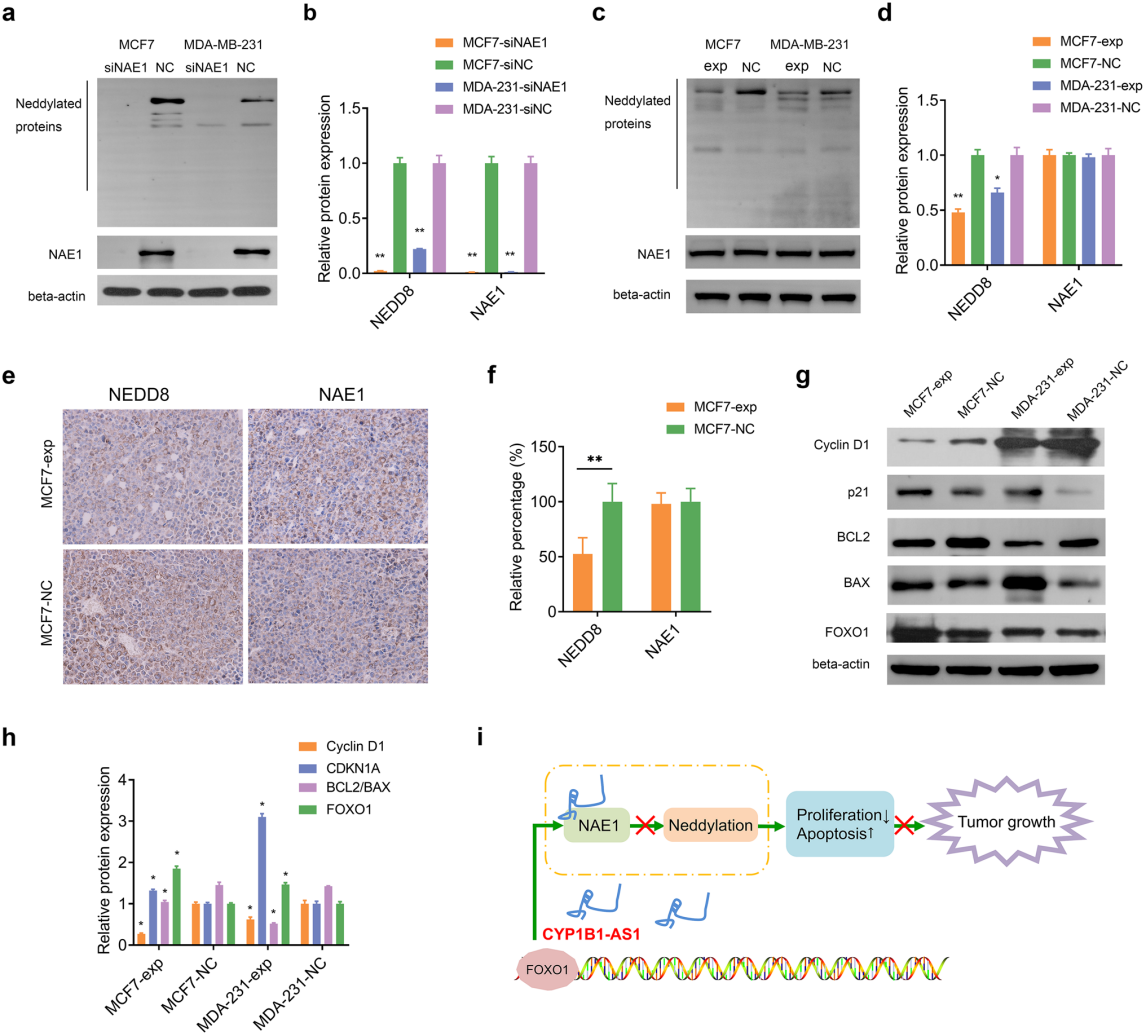
CYP1B1-AS1 affects neddylation through NAE1.** a**, **b** Western blotting was used to detect neddylated proteins in MCF7 and MDA-MB-231 cells after interference with NAE1. siNAE1, NAE1 siRNA. siNC, negative control for siRNA. **P < 0.01. **c**, **d** Western blotting was used to detect NAE1 and neddylated proteins in MCF7 and MDA-MB-231 cells after CYP1B1-AS1 upregulation. *P < 0.05, **P < 0.01.** e**, **f** Representative images of immunohistochemical staining showing the expression of NEDD8 and NAE1 in xenograft tumors. **P < 0.01. **g**, **h** Western blot was used to detect the changes in cyclin D1, p21, BCL2, BAX and FOXO1 after up-regulation of CYP1B1-AS1. *P < 0.05. **i** Schematic diagram of lncRNA CYP1B1-AS1 inhibiting the proliferation of breast cancer by binding NAE1

We used siRNA to silence NAE1 expression and found that neddylated proteins were significantly decreased in MCF7 and MDA-MB-231 cells (Fig. 6a, b). We then examined the total protein in MCF7-exp and MDA-231-exp cells and found decreased neddylated proteins; suggesting that the binding of CYP1B1-AS1 to NAE1 partially inhibited NEDD8 activation (Fig. 6c, d). Interestingly, transcriptome sequencing data and Western blot results did not support a regulatory role for CYP1B1-AS1 on NAE1 expression. Immunohistochemical analysis of xenografts in nude mice also showed that up-regulation of CYP1B1-AS1 caused a decrease in neddylated proteins, but no significant change in NAE1 expression (Fig. 6e, f). We examined several downstream functional proteins regulated by neddylation and showed that CYP1B1-AS1 down-regulated cyclin D1, up-regulated p21 and FOXO1, and decreased BCL2/BAX values (Fig. 6g, h). Given that NAE is a heterodimer formed by NAE1 and ubiquitin-like modification activating enzyme 3 (UBA3) [18], we performed co-immunoprecipitation and showed that UBA3 binding to NAE1 was reduced in MCF7-exp cells (Fig. S3).

## Discussion

Recent studies have highlighted that hyperactivated neddylation disrupts intracellular protein homeostasis and becomes a common event in the development of breast cancer [19, 20]. However, the regulatory mechanisms of neddylation have not been fully explored. In this study, we found that CYP1B1-AS1 was significantly downregulated in breast cancer, showing potential as a tumor diagnostic molecule and prognostic factor. In vivo and in vitro functional experiments showed that CYP1B1-AS1 inhibited cell proliferation and induced apoptosis. Mechanistically, FOXO1-regulated CYP1B1-AS1 affected the heterodimeric enzyme NAE by directly binding to NAE1 to regulate protein neddylation, thereby inhibiting the malignant progression of breast cancer (Fig. 6i).

Although lncRNAs are less conserved, their promoter sequences are similarly conserved to mRNAs, underscoring the importance of transcription factors in the regulation of lncRNA expression [21]. FOXO1 belongs to the forkhead transcription factor family, which can act as a tumor suppressor to regulate the expression of various genes to control important processes such as cell proliferation, survival and resistance to oxidative stress [22]. FOXO1-regulated lncRNAs have been reported in some studies [23, 24]. Our results show that FOXO1 binds to the CYP1B1-AS1 promoter sequence and positively regulates its expression in breast cancer cells. FOXO1 is easily phosphorylated by active PI3K signaling pathway and eventually hydrolyzed by the proteasome [25, 26]. Our data suggest that the PI3K inhibitor LY294002 can effectively reverse FOXO1 protein levels and upregulate CYP1B1-AS1 expression. Therefore, we conclude that CYP1B1-AS1 is regulated by the transcription factor FOXO1 and can be upregulated by inhibiting the PI3K/FOXO1 pathway.

NAE1 is an important subunit of NAE enzyme, which is involved in regulating the turnover of proteasome upstream protein subsets [27]. Our study showed that CYP1B1-AS1 binds stably to NAE1, and this binding reduced intracellular neddylated protein, which did not appear to be achieved by affecting the expression of NAE1 protein. Direct binding of lncRNAs to proteins can trigger changes in protein structure, activity, and function [28]. For example, lnc-Lsm3b can competitively bind to RIG-I monomers and prevent downstream signaling by restricting the conformational transition of its protein [29]. lncRNA-ACOD1 directly binds the metabolic enzyme GOT2 near the substrate niche and enhances its catalytic activity [30]. Since upregulation of CYP1B1-AS1 significantly reduced the amount of UBA3 co-precipitated with NAE1, we speculated that CYP1B1-AS1 binding to NAE1 could block the formation of NAE heterodimer, and more studies are needed here.

NEDD8 regulates the balance between protein "quality" and "quantity" by binding to substrate proteins [31]. The most typical substrates of NEDD8 are the cullin family [32]. Cullins act as scaffolding proteins to recruit adaptor proteins, substrate receptors, and RING proteins to form a multi-unit cullin-RING E3 ligase (CRLs) that mediate approximately 20% of protein ubiquitination hydrolysis [33]. The coupling of NEDD8 to the C-terminal lysine residue of cullins promotes the assembly of functional CRLs, which is essential for CRLs activation [34]. In addition, several non-cullins substrates [35], such as p53 [36] and von Hippel–Lindau tumor suppressor [37], were also identified. Substrate properties determine the role of neddylation in regulating biological processes and disease management [38]. We examined several proteins known to be regulated by neddylation: tumor suppressor protein p21 [23], cell cycle regulator protein Cyclin D1 [39], apoptosis-related proteins BCL2 and BAX [40], and the results showed that these proteins have corresponding expression changes with the upregulation of CYP1B1-AS1. We propose that impaired neddylation triggers the accumulation of CRLs and other NEDD8 substrates, which in turn changes in downstream associated proteins. Notably, FOXO1 is degraded by the proteasome under the action of the CRL1 E3 ubiquitin ligase SCF^Skp2^ [41]. We detected increased FOXO1 protein in cells that upregulated CYP1B1-AS1. Given that transcriptome sequencing data showed no change in FOXO1 mRNA after CYP1B1-AS1 upregulation, we believe that CYP1B1-AS1 enhances FOXO1 protein stability by inhibiting neddylation, thus forming a positive feedback to FOXO1.

Little is known about the role of lncRNAs in affecting neddylation modification. Our study revealed the expression characteristics and biological functions of CYP1B1-AS1 in breast cancer, and proposed the molecular mechanism of CYP1B1-AS1 binding to NAE1 to inhibit neddylation. Upregulating the expression of CYP1B1-AS1 to inhibit neddylation may represent a potential therapeutic strategy for breast cancer.

## Supplementary Information

Below is the link to the electronic supplementary material.

**Supplementary file 1. Table S1**: Primer sequences used in qPCR. **Table S2**: CYP1B1-AS1 probe sequence used in FISH experiment. **Table S3**: siRNAs and negative control. **Table S4**: Primer sequences used in CHIP and dual-luciferase assay. **Table S5**: Primer sequences used in RNA pull-down experiment. **Table S6**: The antibodies used in this study. Data 1 CYP1B1-AS1 lentiviral expression vector sequence. Data 2 FOXO1 expression vector sequence. **Fig. S1**: Expression characteristics of CYP1B1-AS1. **Fig. S2**: Analysis of CYP1B1-AS1 expression regulation. **Fig. S3**: The results of the co-immunoprecipitation assay. **Fig. S4**: Raw data of Western blot. (PDF 2987 kb)

## Data Availability

The lncRNA microarray data have been submitted to the NCBI GEO database under accession number GSE11527547 (https://identifiers.org/geo:GSE115275). The RNA-seq data have been submitted to the NCBI SRA database under accession number PRJNA889988 (https://www.ncbi.nlm.nih.gov/bioproject/889988). The mass spectrometry results of RNA pull-down protein solutions have been submitted to the PRIDE database and data are available via ProteomeXchange with identifier PXD037317.

## Abbreviations

CYP1B1-AS1: LncRNA cytochrome P450 family 1 subfamily B member 1 antisense RNA 1
FOXO1: Forkhead box O1
lncRNAs: Long non-coding RNAs
NAE1: NEDD8 activating enzyme E1 subunit 1
NEDD8: Neural precursor cell expressed developmentally downregulated protein 8
PTM: Post-translational modification
UBA3: Ubiquitin-like modification activating enzyme 3
