# Supplementary material for: FOXO1-regulated lncRNA CYP1B1-AS1 suppresses breast cancer cell proliferation by inhibiting neddylation
Source: Breast Cancer Res Treat. 2023 Aug 28;202(2):397–408. doi: 10.1007/s10549-023-07090-z (PMC10505597; doi:10.1007/s10549-023-07090-z)
Supplement: Supplementary file 1 — Table S1: Primer sequences used in qPCR. Table S2: CYP1B1-AS1 probe sequence used in FISH experiment. Table S3: siRNAs and negative control. Table S4: Primer sequences used in CHIP and dual-luciferase assay. Table S5: Primer sequences used in RNA pull-down experiment. Table S6: The antibodies used in this study. Data 1 CYP1B1-AS1 lentiviral expression vector sequence. Data 2 FOXO1 expression vector sequence. Fig. S1: Expression characteristics of CYP1B1-AS1. Fig. S2: Analysis of CYP1B1-AS1 expression regulation. Fig. S3: The results of the co-immunoprecipitation assay. Fig. S4: Raw data of Western blot. (PDF 2987 kb) [file 10549_2023_7090_MOESM1_ESM.pdf]

Supplementary Table 1:

Primer sequences used in qPCR.

Primer	Sequence (5' to 3')
CYP1B1-AS1 Forward	ACACAAGAATCGGCACTGGT
CYP1B1-AS1 Reverse	GCCGCATGTTTCATCTGCAT
FOXO1 Forward	CCGGAGTTTAGCCAGTCCAA
FOXO1 Reverse	CACGCTCTTGACCATCCACT
NAE1 Forward	GGCCAAAGAGGGTCAAGGAA
NAE1 Reverse	AAATGGACTCTGGTGCCTGG
U6 Forward	CTCGCTTCGGCAGCACA
U6 Reverse	AACGCTTCACGAATTTGCGT
GAPDH Forward	CCGGGAAACTGTGGCGTGATGG
GAPDH Reverse	AGGTGGAGGAGTGGGTGTCGCTGTT

Supplementary Table 2:

CYP1B1-AS1 probe sequence used in FISH experiment.

Name	Sequence (5’-3’)
NR-027252.1：	TGTTTCCTAGTTAGTCGCTTGAACTTCCAATGCCCTAT

Supplementary Table 3:

SiRNAs and negative control.

Name	Sequence (5' to 3 ')
FOXO1 siRNA1	CGGGCUGGAAGAAUUCAAUTT
FOXO1 siRNA2	CCCUCGAACUAGCUCAAAUTT
NAE1 siRNA	GGGUUGUGCUUUAGUCUGUTT
Negative control	UUCUCCGAACGUGUCACGUTT

Supplementary Table 4:

Primer sequences used in CHIP and dual-luciferase assay.

Primer	Sequence (5' to 3')
Promoter Forward	GTGACATTATTTATGAACCTGCTGC
Promoter Reverse	TGGGTGCTTCCTGCTGTTG

Supplementary Table 5:

Primer sequences used in RNA pull-down experiment.

Primer	Sequence (5' to 3')
CYP1B1-AS1 sense Forward	taatacgactcactatagggTGGTTGCTAACACAAGAATCG
CYP1B1-AS1 sense Reverse	AGTGAGTTATGGTCACCTTGGAATGG
CYP1B1-AS1 antisense Forward	taatacgactcactatagggAGTGAGTTATGGTCACCTTGGAATGG
CYP1B1-AS1 antisense Reverse	TGGTTGCTAACACAAGAATCG

Supplementary Table 6:

The antibodies used in this study.

**Antibody**	**Specificity**	**Manufacturer**	**Code**
anti-FOXO1	Rabbit monoclonal antibody	CST	2880
anti-NAE1	Rabbit monoclonal antibody	Abcam	ab187142
anti-p21	Rabbit monoclonal antibody	Abcam	Ab109520
anti-cyclin D1	Rabbit monoclonal antibody	Abcam	ab40754
anti-BCL2	Rabbit monoclonal antibody	Abcam	ab32124
anti-BAX	Rabbit monoclonal antibody	Abcam	ab32503
anti-NEDD8	Rabbit monoclonal antibody	Abcam	ab81264
anti- UBA3	Rabbit monoclonal antibody	Abcam	ab124728
anti-beta-actin	Rabbit polyclonal antibody	Abcam	ab8227

```
                 10        20        30        40        50        60        70
         CATACATTATACGAAGTTATATTAAGGGTTCCAAGCTTAAGCGGCCGCCAACTATGGTTGCTAACACAAGAATCGGCA
H2470-2.EF1a-F.ab1(50>700) →
```

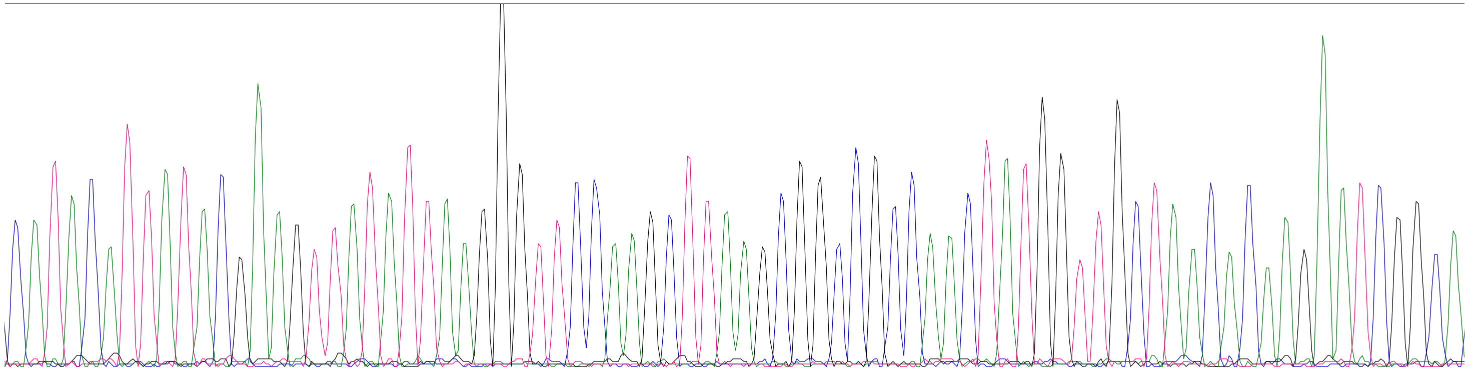

```
         CATACATTATACGAAGTTATATTAAGGGTTCCAAGCTTAAGCGGCCGCCAACTATGGTTGCTAACACAAGAATCGGCA
B9401 Homo CYP1B1-AS1.seq(1>1776) →                        CAACTATGGTTGCTAACACAAGAATCGGCA

          80        90        100       110       120       130       140       150
         CTGGTGACTTTTGAGCCATCAATCATCCTTTCCTAACACATGAATCTTTTGGAAAATGGTCTCAAATAGTCAAGACCC
H2470-2.EF1a-F.ab1(50>700) →
```

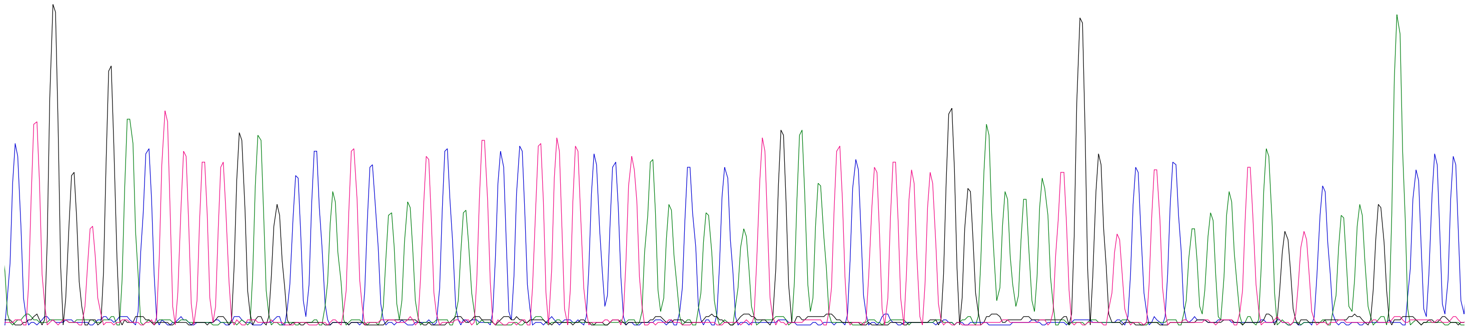

```
         CTGGTGACTTTTGAGCCATCAATCATCCTTTCCTAACACATGAATCTTTTGGAAAATGGTCTCAAATAGTCAAGACCC
B9401 Homo CYP1B1-AS1.seq(1>1776) →
         CTGGTGACTTTTGAGCCATCAATCATCCTTTCCTAACACATGAATCTTTTGGAAAATGGTCTCAAATAGTCAAGACCC

            160       170       180       190       200       210       220       230
         CCTGCTGGGAGGAGCTTCTATCCTGCTCTGTGGAAGAAATGGGCATGCCAGATGCCTGGAAGATCAGAGGCTCACATT
```

Project: Alignment of Homo CYP1B1-AS1.sqd Contig 1

```
                                        160       170       180       190       200       210       220       230
                                        CCTGCTGGGAGGAGCTTCTATCCTGCTCTGTGGAAGAAATGGGCATGCCAGATGCCTGGAAGATCAGAGGCTCACATT
H2470-2.EF1a-F.ab1(50>700)         →
```

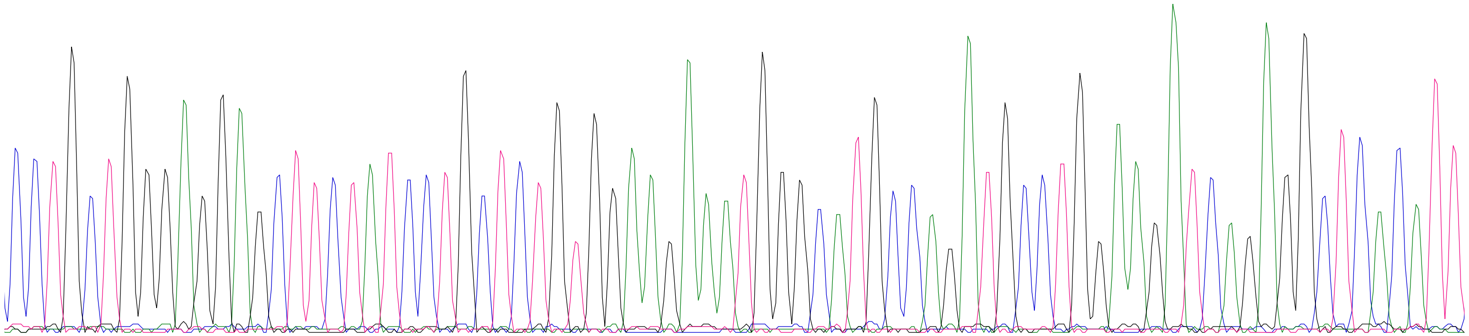

```
                                        CCTGCTGGGAGGAGCTTCTATCCTGCTCTGTGGAAGAAATGGGCATGCCAGATGCCTGGAAGATCAGAGGCTCACATT
B9401 Homo CYP1B1-AS1.seq(1>1776)  →   CCTGCTGGGAGGAGCTTCTATCCTGCTCTGTGGAAGAAATGGGCATGCCAGATGCCTGGAAGATCAGAGGCTCACATT

                                          240       250       260       270       280       290       300       310
                                        CCAGTTCAAAGTTAGAGAAACCTGAGAGGGGTGTACTGGTATGACCACCCGAGATCCCATCGCTGAGAAAGATAATGG
H2470-2.EF1a-F.ab1(50>700)         →
```

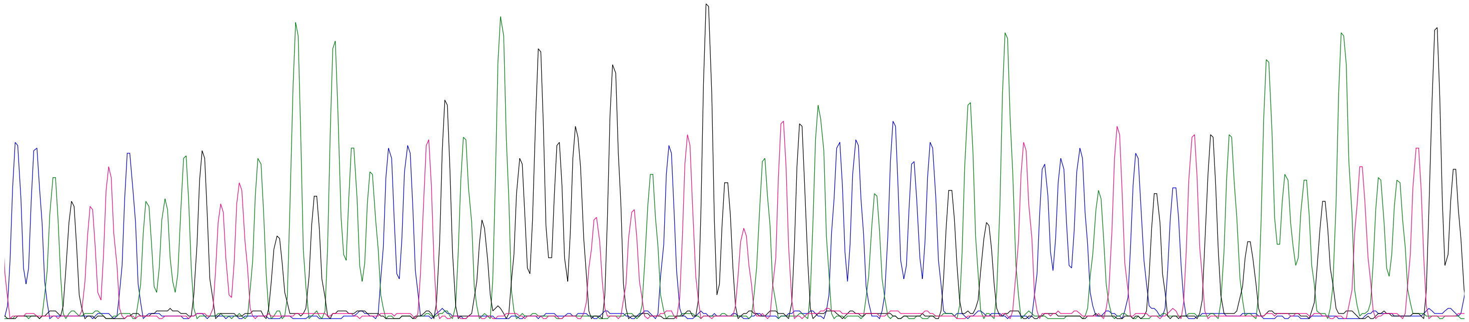

```
                                        CCAGTTCAAAGTTAGAGAAACCTGAGAGGGGTGTACTGGTATGACCACCCGAGATCCCATCGCTGAGAAAGATAATGG
B9401 Homo CYP1B1-AS1.seq(1>1776)  →   CCAGTTCAAAGTTAGAGAAACCTGAGAGGGGTGTACTGGTATGACCACCCGAGATCCCATCGCTGAGAAAGATAATGG

                                            320       330       340       350       360       370       380       390
                                        CCCTGGAGACAAAGACTGATGAAGAAAGACAGTATCCAGCTGTGATGCAGATGAAACATGCGGCATGGTTGCACTCAC
```

320 330 340 350 360 370 380 390

CCCTGGAGACAAAGACTGATGAAGAAAGACAGTATCCAGCTGTGATGCAGATGAAACATGCGGCATGGTTGCACTCAC

H2470-2.EF1a-F.ab1(50>700) →

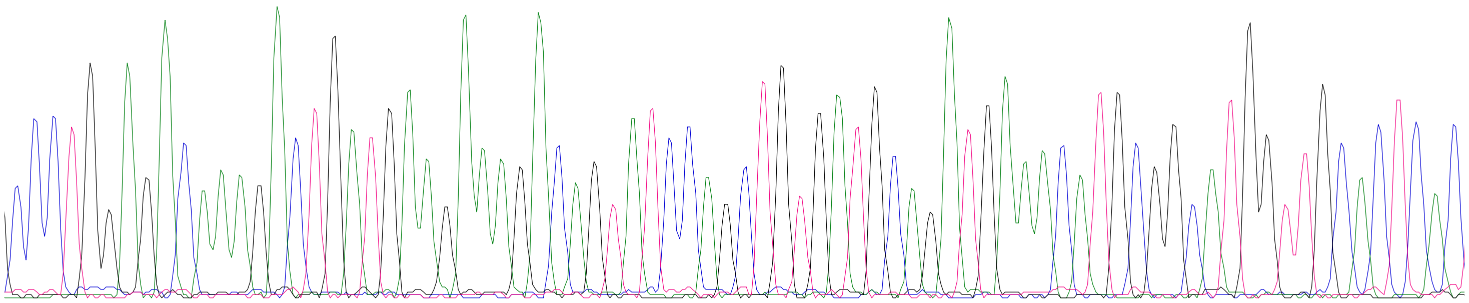

CCCTGGAGACAAAGACTGATGAAGAAAGACAGTATCCAGCTGTGATGCAGATGAAACATGCGGCATGGTTGCACTCAC

B9401 Homo CYP1B1-AS1.seq(1>1776) → CCCTGGAGACAAAGACTGATGAAGAAAGACAGTATCCAGCTGTGATGCAGATGAAACATGCGGCATGGTTGCACTCAC

400 410 420 430 440 450 460

TATGCAGCCAAGGCTGGAAGCCACTCTGGATCCAGCCACTCTGGCACAGCGTTCTTGCTCTAGGAAGAACAACTGAAC

H2470-2.EF1a-F.ab1(50>700) →

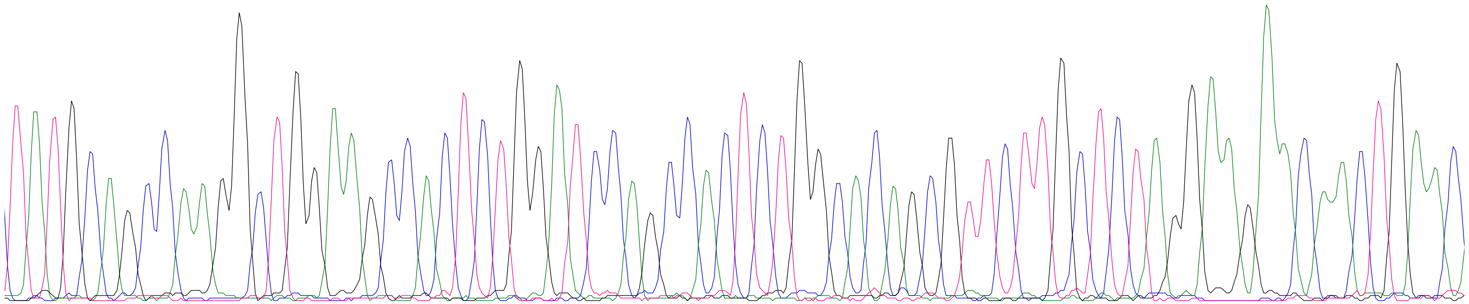

TATGCAGCCAAGGCTGGAAGCCACTCTGGATCCAGCCACTCTGGCACAGCGTTCTTGCTCTAGGAAGAACAACTGAAC

B9401 Homo CYP1B1-AS1.seq(1>1776) → TATGCAGCCAAGGCTGGAAGCCACTCTGGATCCAGCCACTCTGGCACAGCGTTCTTGCTCTAGGAAGAACAACTGAAC

470 480 490 500 510 520 530 540

ATGGCCACACAGGCCTCACAGCTGGCATATGGAGGCTGTTAATAAGCTGAGCTTGAGGCTTCAAAGGACTCTTGTAGC

470 480 490 500 510 520 530 540

ATGGCCACACAGGCCTCACAGCTGGCATATGGAGGCTGTTAATAAGCTGAGCTTGAGGCTTCAAAGGACTCTTGTAGC

H2470-2.EF1a-F.ab1(50>700) →

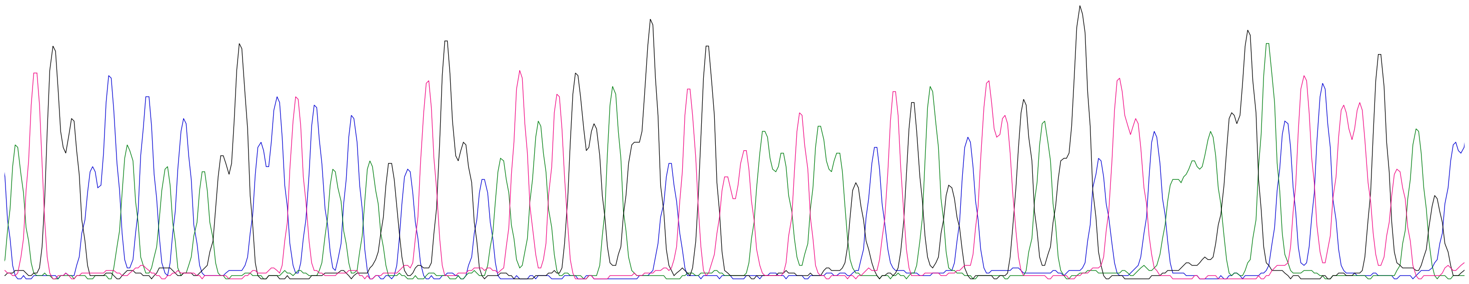

ATGGCCACACAGGCCTCACAGCTGGCATATGGAGGCTGTTAATAAGCTGAGCTTGAGGCTTCAAAGGACTCTTGTAGC

B9401 Homo CYP1B1-AS1.seq(1>1776) → ATGGCCACACAGGCCTCACAGCTGGCATATGGAGGCTGTTAATAAGCTGAGCTTGAGGCTTCAAAGGACTCTTGTAGC

550 560 570 580 590 600 610 620

CTGTGCTGGATCTAGCAGTGACCAGTATGCCAGTGGCCATGGATGCCCAGGCCCATGTTCAGTAGTGAAGCAGGTGCT

H2470-2.EF1a-F.ab1(50>700) →

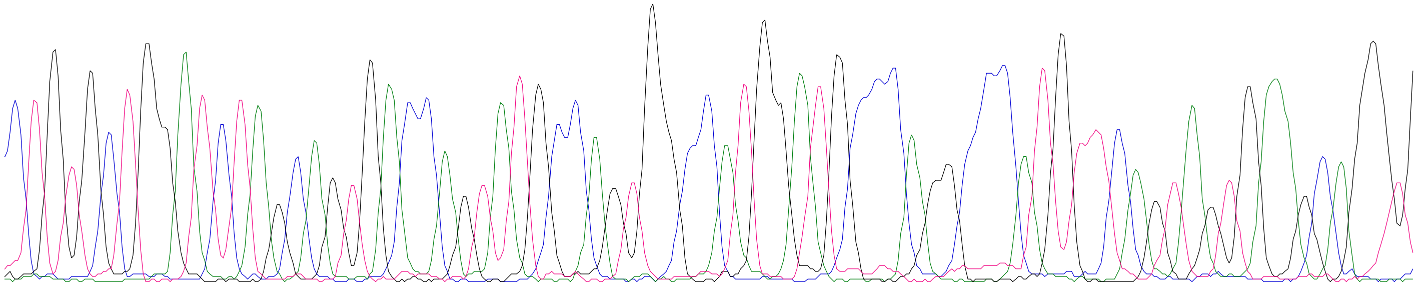

CTGTGCTGGATCTAGCAGTGACCAGTATGCCAGTGGCCATGGATGCCCAGGCCCATGTTCAGTAGTGAAGCAGGT

B9401 Homo CYP1B1-AS1.seq(1>1776) → CTGTGCTGGATCTAGCAGTGACCAGTATGCCAGTGGCCATGGATGCCCAGGCCCATGTTCAGTAGTGAAGCAGGTGCT

550 560 570 580 590 600 610 620

CTGTGCTGGATCTAGCAGTGACCAGTATGCCAGTGGCCATGGATGCCCAGGCCCATGTTCAGTAGTGAAGCAGGTGCT

H2470-2.B9401F1.ab1(50>700) →

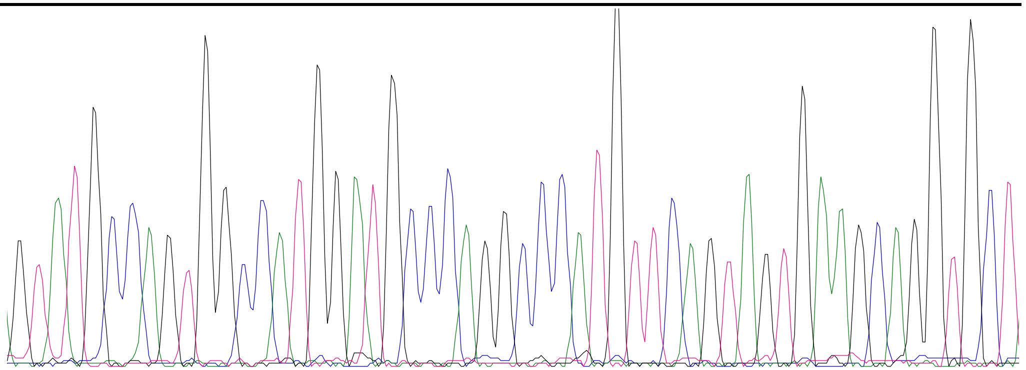

GTATGCCAGTGGCCATGGATGCCCAGGCCCATGTTCAGTAGTGAAGCAGGTGCT

630 640 650 660 670 680 690 700

AGCAGCAGAAGCAATAGAAATGGAAACTGGGAGATCTGCTGTGGATGAATCTCATTATCTTCTTTCTCTGACCCTCTT

B9401 Homo CYP1B1-AS1.seq(1>1776) → AGCAGCAGAAGCAATAGAAATGGAAACTGGGAGATCTGCTGTGGATGAATCTCATTATCTTCTTTCTCTGACCCTCTT

H2470-2.B9401F1.ab1(50>700) →

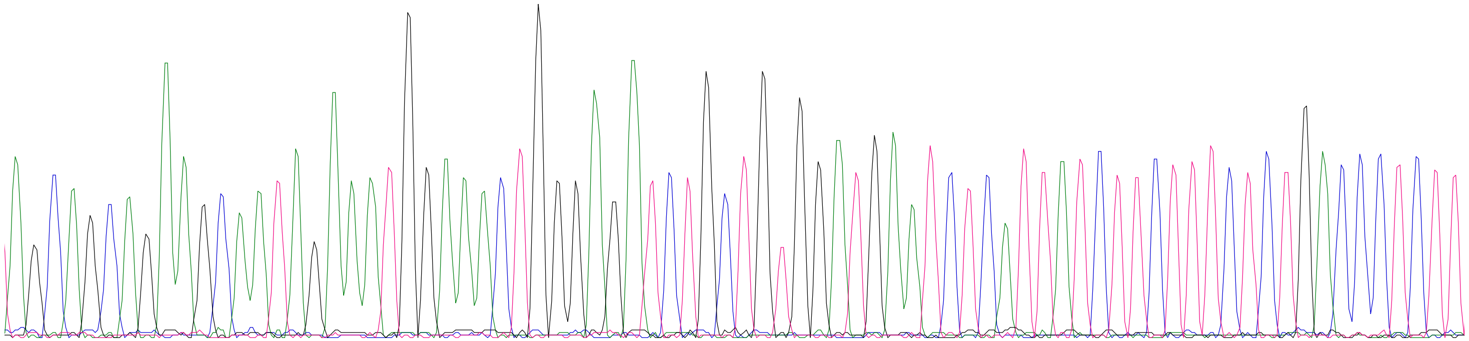

AGCAGCAGAAGCAATAGAAATGGAAACTGGGAGATCTGCTGTGGATGAATCTCATTATCTTCTTTCTCTGACCCTCTT

```
                                              710       720       730       740       750       760       770       780
                                            TGGACTAAATAAACTCCCAGTATTCTTGACCAACAGAGGTAGGGTTGGAACAGAACCTCAAAAGTCATCATTAGACTT
B9401 Homo CYP1B1-AS1.seq(1>1776)      →    TGGACTAAATAAACTCCCAGTATTCTTGACCAACAGAGGTAGGGTTGGAACAGAACCTCAAAAGTCATCATTAGACTT
H2470-2.B9401F1.ab1(50>700)            →
```

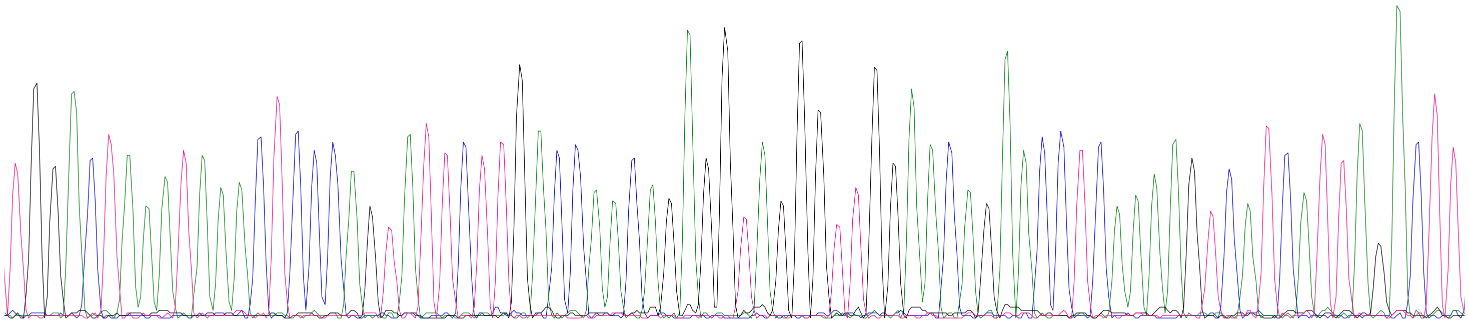

```
TGGACTAAATAAACTCCCAGTATTCTTGACCAACAGAGGTAGGGTTGGAACAGAACCTCAAAAGTCATCATTAGACTT
```

```
                                                 790       800       810       820       830       840       850
                                            CCTACTAATAGGGCATTGGAAGTTCAAGCGACTAACTAGGAAACAGGTTAGTATTTGTACCCCTACACTGTGCAACAG
B9401 Homo CYP1B1-AS1.seq(1>1776)      →    CCTACTAATAGGGCATTGGAAGTTCAAGCGACTAACTAGGAAACAGGTTAGTATTTGTACCCCTACACTGTGCAACAG
H2470-2.B9401F1.ab1(50>700)            →
```

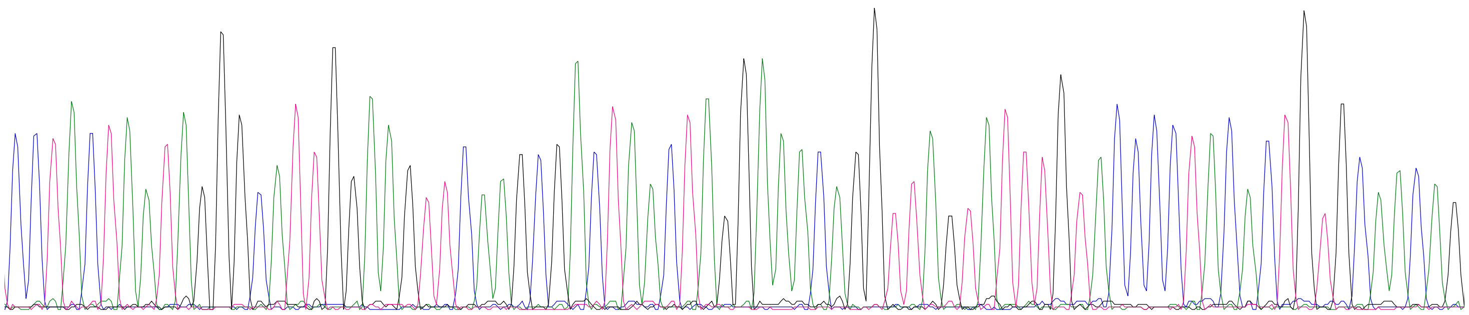

```
CCTACTAATAGGGCATTGGAAGTTCAAGCGACTAACTAGGAAACAGGTTAGTATTTGTACCCCTACACTGTGCAACAG
```

860 870 880 890 900 910 920 930

ATTACATACCATTTGCTCTATTTGGCATCCTTTCCTTAGGCTAGATTCCCAGAAGAAAGATTACTGATTCGAAGGGCA

```
B9401 Homo CYP1B1-AS1.seq(1>1776)  →  ATTACATACCATTTGCTCTATTTGGCATCCTTTCCTTAGGCTAGATTCCCAGAAGAAAGATTACTGATTCGAAGGGCA
H2470-2.B9401F1.ab1(50>700)        →
```

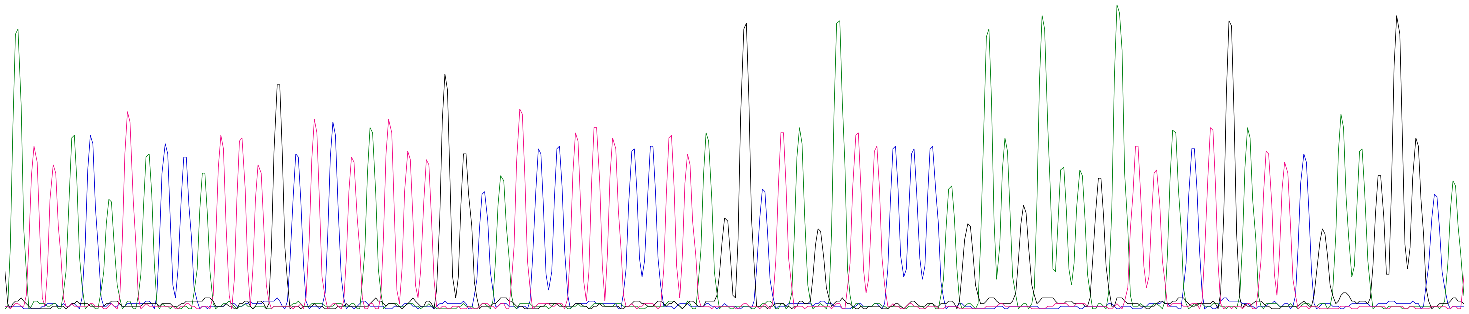

ATTACATACCATTTGCTCTATTTGGCATCCTTTCCTTAGGCTAGATTCCCAGAAGAAAGATTACTGATTCGAAGGGCA

940 950 960 970 980 990 1000 1010

TGAACATTTTTATGACTCTTGGCCTAAATTACTAGGCCAAGGTTTTTTCAAAGGGATGCACTAATTTTCACTATTTCC

```
B9401 Homo CYP1B1-AS1.seq(1>1776)  →  TGAACATTTTTATGACTCTTGGCCTAAATTACTAGGCCAAGGTTTTTTCAAAGGGATGCACTAATTTTCACTATTTCC
H2470-2.B9401F1.ab1(50>700)        →
```

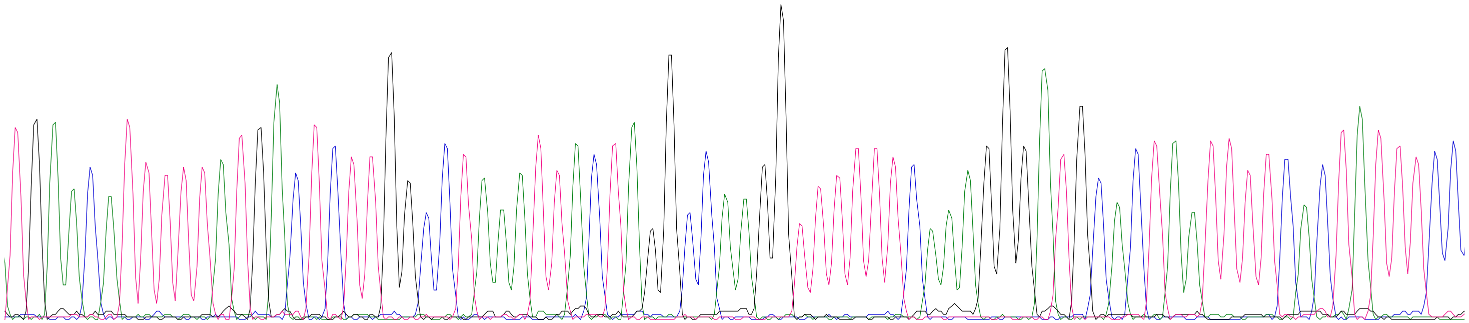

TGAACATTTTTATGACTCTTGGCCTAAATTACTAGGCCAAGGTTTTTTCAAAGGGATGCACTAATTTTCACTATTTCC

	1020 1030 1040 1050 1060 1070 1080 1090
	CCACACCCTTTCAGGTGATAAATAATTGACTTGATTTGTTTACACTGACGCCAAGAATTCTTGGGAAGAAGGATCAAA
B9401 Homo CYP1B1-AS1.seq(1>1776) →	CCACACCCTTTCAGGTGATAAATAATTGACTTGATTTGTTTACACTGACGCCAAGAATTCTTGGGAAGAAGGATCAAA
H2470-2.B9401F1.ab1(50>700) →	

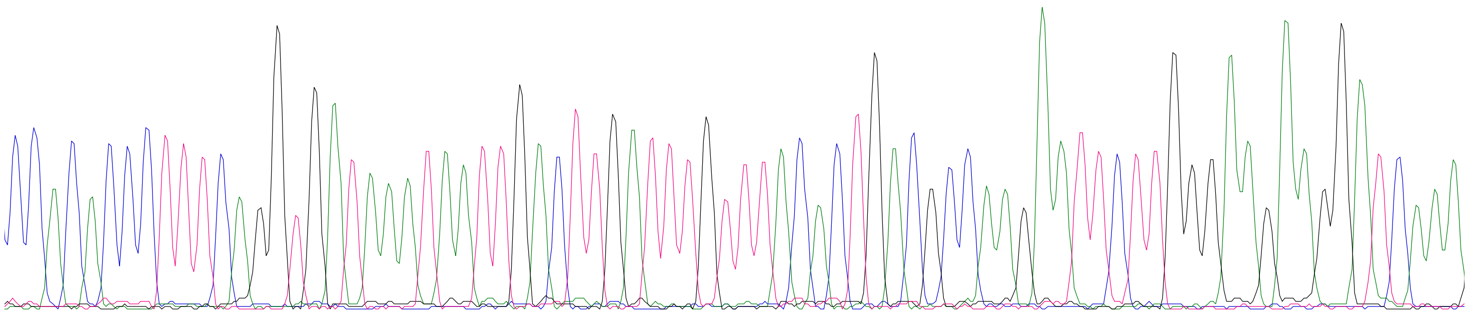

CCACACCCTTTCAGGTGATAAATAATTGACTTGATTTGTTTACACTGACGCCAAGAATTCTTGGGAAGAAGGATCAAA

	1100 1110 1120 1130 1140 1150 1160 1170
	GTGAAAAGTGGTAGCCCATGGTGGGGAGGCCACACTCAGTGCAGTTGTGAAGTCAGCAGCATTTTTAAGCCCATTCAT
B9401 Homo CYP1B1-AS1.seq(1>1776) →	GTGAAAAGTGGTAGCCCATGGTGGGGAGGCCACACTCAGTGCAGTTGTGAAGTCAGCAGCATTTTTAAGCCCATTCAT
H2470-2.B9401F1.ab1(50>700) →	

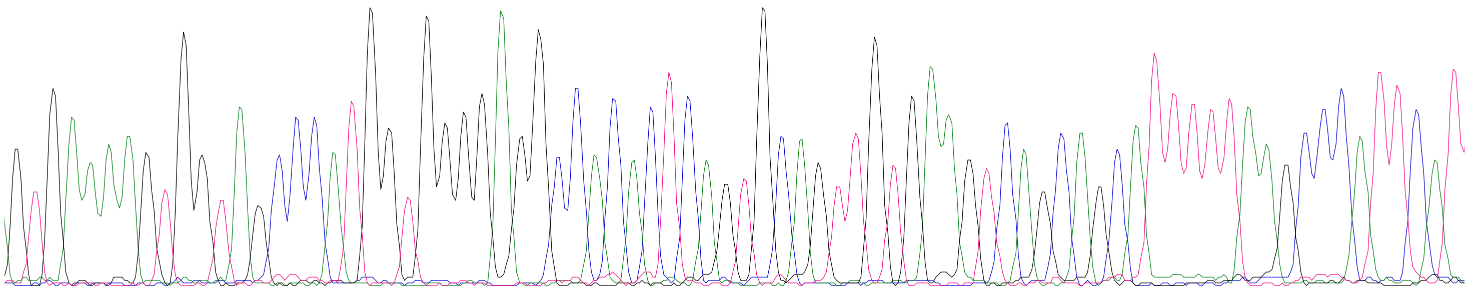

GTGAAAAGTGGTAGCCCATGGTGGGGAGGCCACACTCAGTGCAGTTGTGAAGTCAGCAGCATTTTTAAGCCCATTCAT

```
                                              1180      1190      1200      1210      1220      1230      1240
                                          TGCCAGTAGCCCTGAGATGAGCATTCCCAAGCTGCTTCCCAGGTCCCTCCTCCACTCTCTCAGGGACATCCATTCTGT
B9401 Homo CYP1B1-AS1.seq(1>1776)     →   TGCCAGTAGCCCTGAGATGAGCATTCCCAAGCTGCTTCCCAGGTCCCTCCTCCACTCTCTCAGGGACATCCATTCTGT
H2470-2.B9401F1.ab1(50>700)           →
```

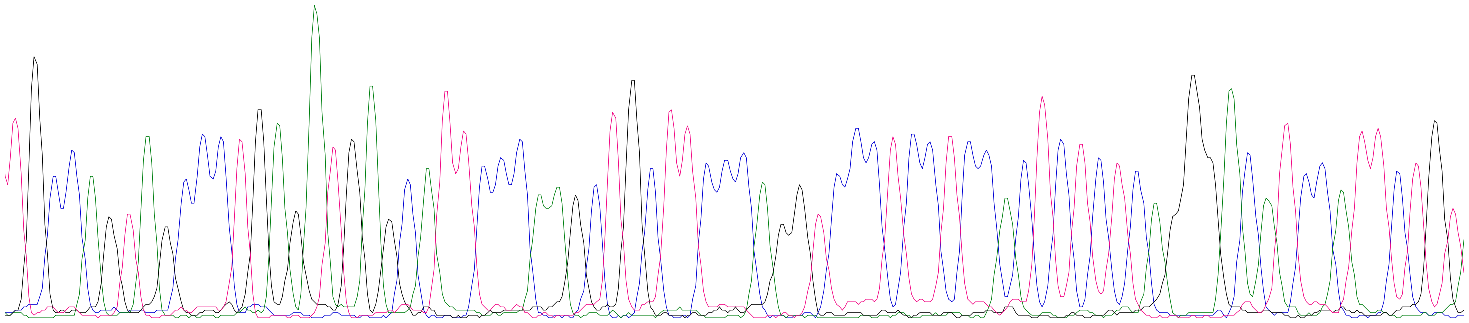

```
                                          TGCCAGTAGCCCTGAGATGAGCATTCCCAAGCTGCTTCCCAGGTCCCTCCTCCACTCTCTCAGGGACATCCATTCTGT
```

```
                                        1250      1260      1270      1280      1290      1300      1310      1320
                                          ACCAGCCCCCTCAGATACTCATGCCCTTGCCCTCCCTTCTCCATGCTCATTAAAACCAGCTTAAGAGCAGATATGACA
B9401 Homo CYP1B1-AS1.seq(1>1776)     →   ACCAGCCCCCTCAGATACTCATGCCCTTGCCCTCCCTTCTCCATGCTCATTAAAACCAGCTTAAGAGCAGATATGACA
H2470-2.B9401F1.ab1(50>700)           →
```

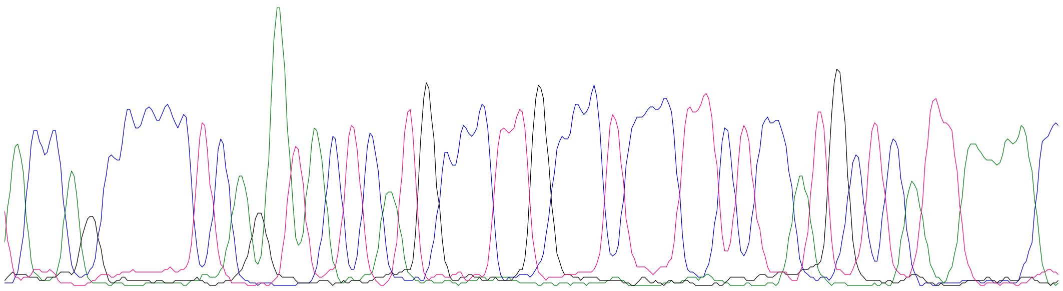

```
                                          ACCAGCCCCCTCAGATACTCATGCCCTTGCCCTCCCTTCTCCATGCTCATTAAAAC
```

1250 1260 1270 1280 1290 1300 1310 1320

ACCAGCCCCCTCAGATACTCATGCCCTTGCCCTCCCTTCTCCATGCTCATTAAAACCAGCTTAAGAGCAGATATGACA

H2470-2.B9401F2.ab1(50>700) →

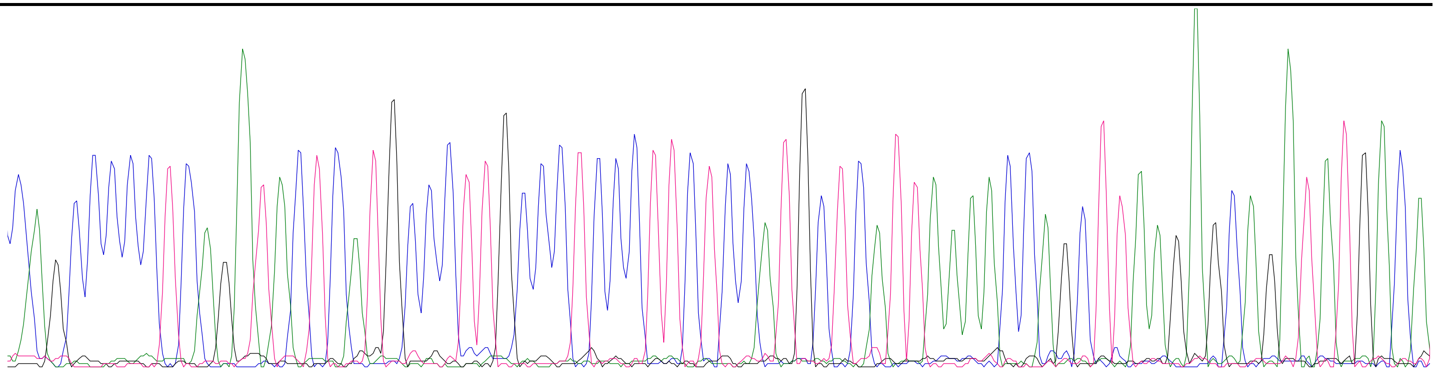

CAGCCCCCTCAGATACTCATGCCCTTGCCCTCCCTTCTCCATGCTCATTAAAACCAGCTTAAGAGCAGATATGACA

1330 1340 1350 1360 1370 1380 1390 1400

TCAGTCACTTACCTTTTCGTGTGGCTCTAAATCAAAGTGTCACCTCTCCCAAGCACATGCTTGCATCTTAGCTCTGAT

B9401 Homo CYP1B1-AS1.seq(1>1776) → TCAGTCACTTACCTTTTCGTGTGGCTCTAAATCAAAGTGTCACCTCTCCCAAGCACATGCTTGCATCTTAGCTCTGAT

H2470-2.B9401F2.ab1(50>700) →

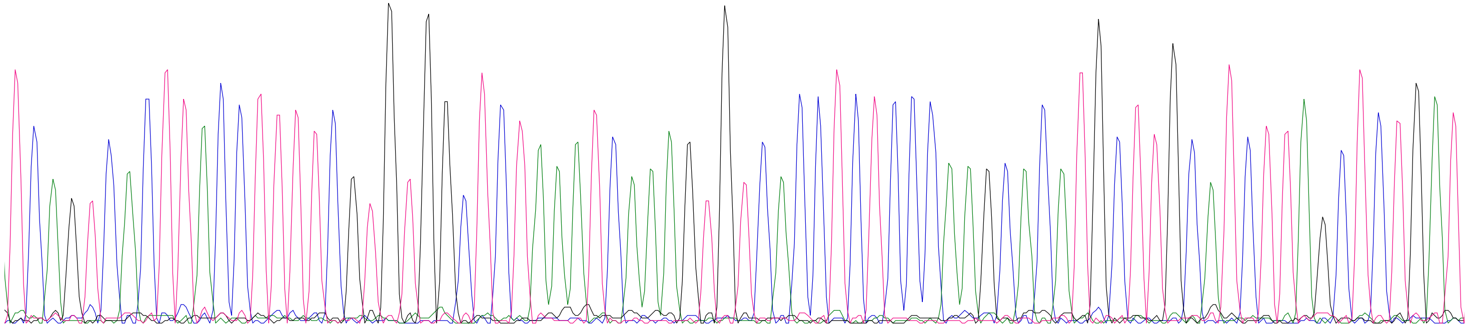

TCAGTCACTTACCTTTTCGTGTGGCTCTAAATCAAAGTGTCACCTCTCCCAAGCACATGCTTGCATCTTAGCTCTGAT

```
                                         1410      1420      1430      1440      1450      1460      1470      1480
                                         TGAGGAATGAAAAGCTTTGTTCTCTGTTAAAAGAACTTTGAGCTTAGATGCCCCAGACTGAGAAATCCACCAAAAGCT
B9401 Homo CYP1B1-AS1.seq(1>1776)    →   TGAGGAATGAAAAGCTTTGTTCTCTGTTAAAAGAACTTTGAGCTTAGATGCCCCAGACTGAGAAATCCACCAAAAGCT
H2470-2.B9401F2.ab1(50>700)          →
```

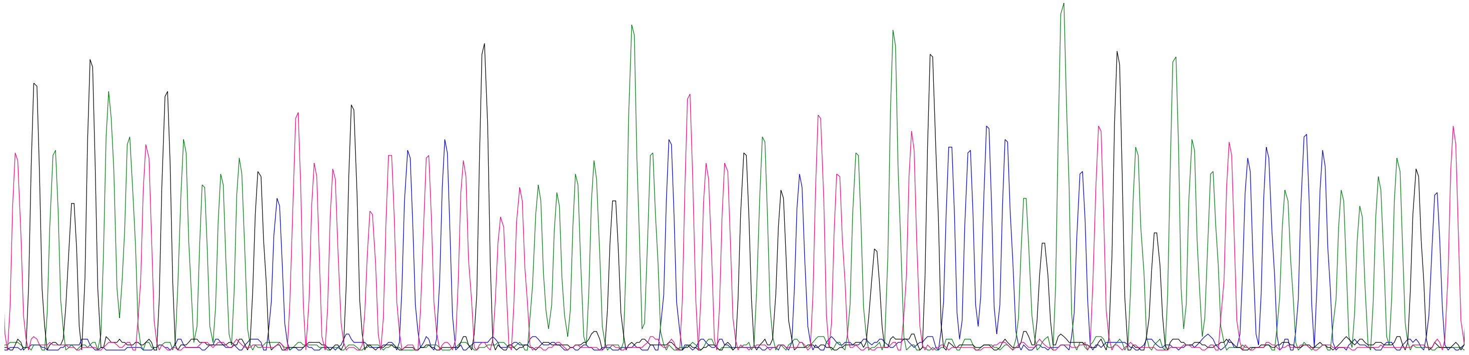

```
                                         TGAGGAATGAAAAGCTTTGTTCTCTGTTAAAAGAACTTTGAGCTTAGATGCCCCAGACTGAGAAATCCACCAAAAGCT
```

```
                                              1490      1500      1510      1520      1530      1540      1550      1560
                                         GGACCTTTGACTTGAGCGGCAAAGGACCCCCATAGTCAGGCTGTGATTTGAGGAGCATCAAGAGGCCAGAGTTTCCAA
B9401 Homo CYP1B1-AS1.seq(1>1776)    →   GGACCTTTGACTTGAGCGGCAAAGGACCCCCATAGTCAGGCTGTGATTTGAGGAGCATCAAGAGGCCAGAGTTTCCAA
H2470-2.B9401F2.ab1(50>700)          →
```

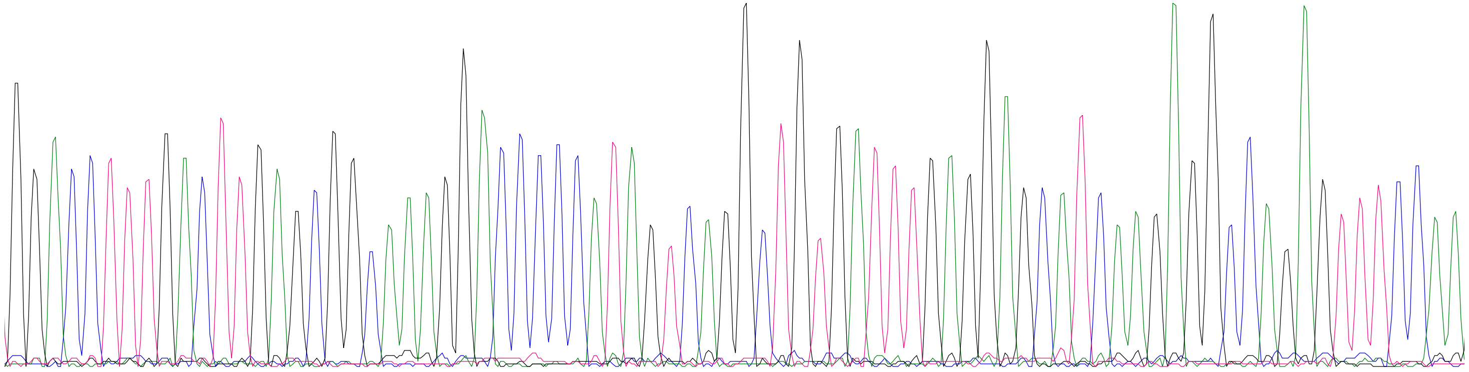

```
                                         GGACCTTTGACTTGAGCGGCAAAGGACCCCCATAGTCAGGCTGTGATTTGAGGAGCATCAAGAGGCCAGAGTTTCCAA
```

```
                                    1570      1580      1590      1600      1610      1620      1630
                                    GTTGGGACCCAGAAGGTGGGGTTGGCAGGGGGAGGGTAGGAGCAGCTGGAGACTCTGCTCTACCAGGGGAATACGGAG
B9401 Homo CYP1B1-AS1.seq(1>1776)   →  GTTGGGACCCAGAAGGTGGGGTTGGCAGGGGGAGGGTAGGAGCAGCTGGAGACTCTGCTCTACCAGGGGAATACGGAG
H2470-2.B9401F2.ab1(50>700)        →
```

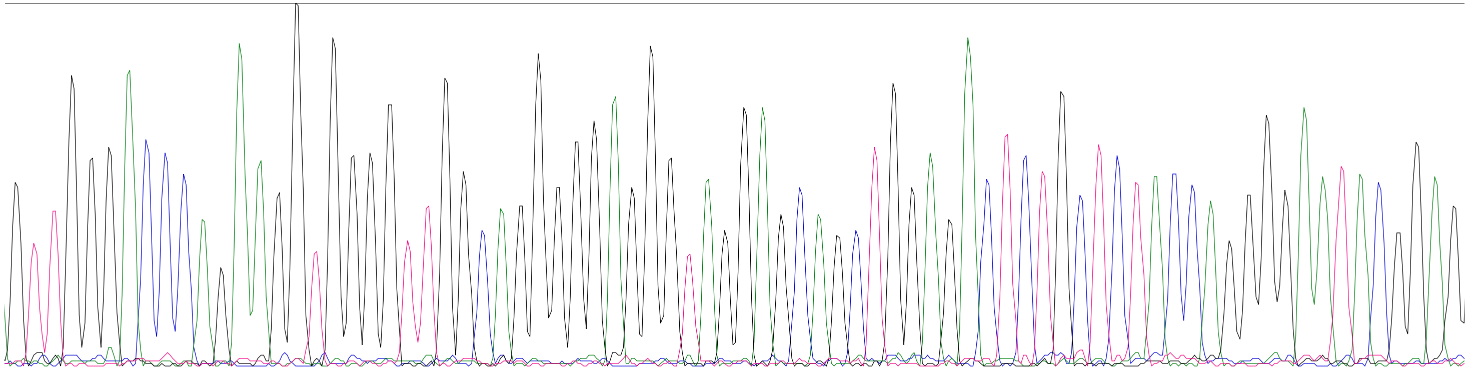

```
GTTGGGACCCAGAAGGTGGGGTTGGCAGGGGGAGGGTAGGAGCAGCTGGAGACTCTGCTCTACCAGGGGAATACGGAG
```

```
                                  1640      1650      1660      1670      1680      1690      1700      1710
                                    GTGGGGACCAATCCCAGGGTAAGGAATGAAAGTAGGAGCCCCAGAAGCTGAAAACATACTTCACCGATGTCAGCATTT
B9401 Homo CYP1B1-AS1.seq(1>1776)   →  GTGGGGACCAATCCCAGGGTAAGGAATGAAAGTAGGAGCCCCAGAAGCTGAAAACATACTTCACCGATGTCAGCATTT
H2470-2.B9401F2.ab1(50>700)        →
```

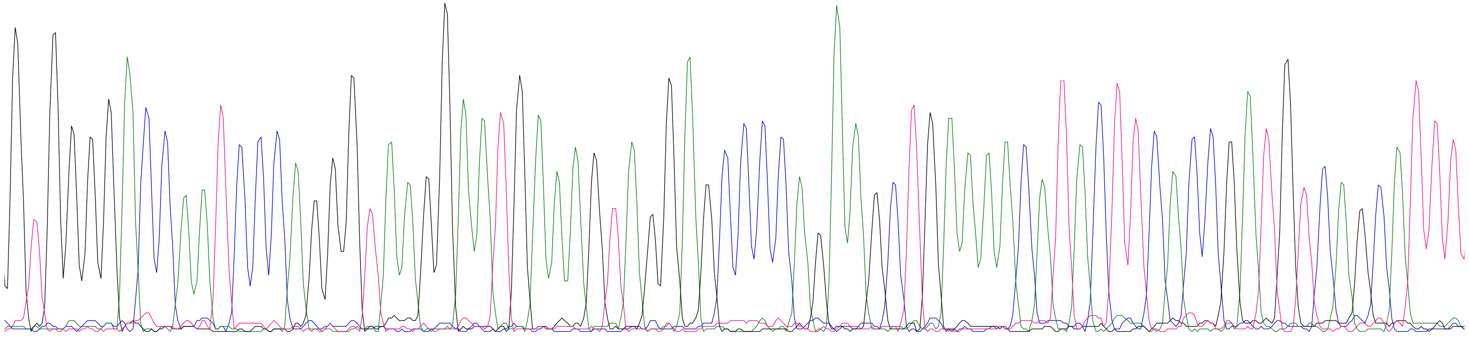

```
GTGGGGACCAATCCCAGGGTAAGGAATGAAAGTAGGAGCCCCAGAAGCTGAAAACATACTTCACCGATGTCAGCATTT
```

1720 1730 1740 1750 1760 1770 1780 1790

TACCCAGAGCCATTCCAAGGTGACCATAACTCACTTAAAAGCCTGAAAGCTGTTTTATGCCAACTAATAAAGTGCAGT

B9401 Homo CYP1B1-AS1.seq(1>1776) → TACCCAGAGCCATTCCAAGGTGACCATAACTCACTTAAAAGCCTGAAAGCTGTTTTATGCCAACTAATAAAGTGCAGT
H2470-2.B9401F2.ab1(50>700) →

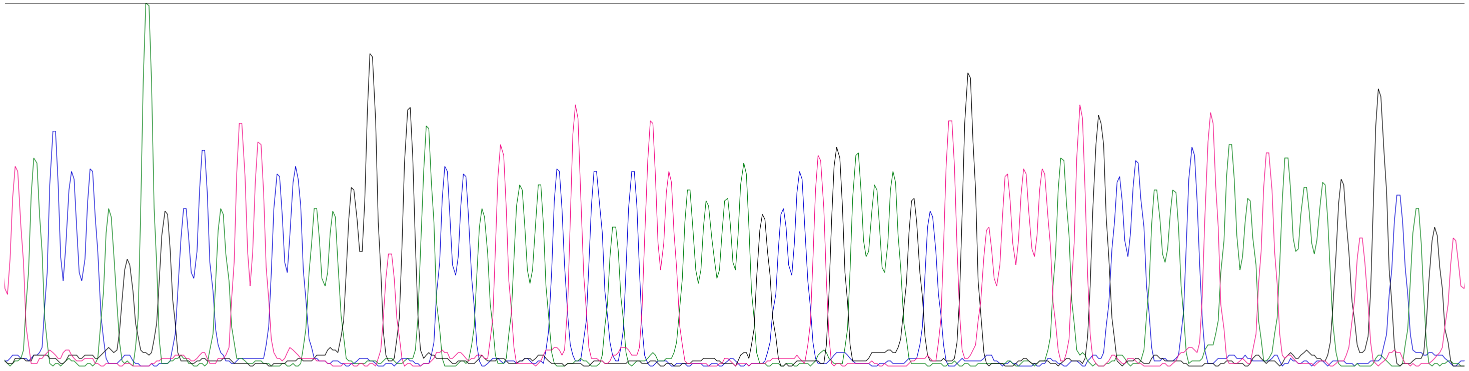

TACCCAGAGCCATTCCAAGGTGACCATAACTCACTTAAAAGCCTGAAAGCTGTTTTATGCCAACTAATAAAGTGCAGT

1800 1810 1820 1830 1840 1850

TTAATAAAAAAAAAAAAAAAAAAAAAAAAATGCATCTACCTAGGGATGGATCCGACACTCTC

B9401 Homo CYP1B1-AS1.seq(1>1776) → TTAATAAAAAAAAAAAAAAAAAAAAAAAAA
H2470-2.B9401F2.ab1(50>700) →

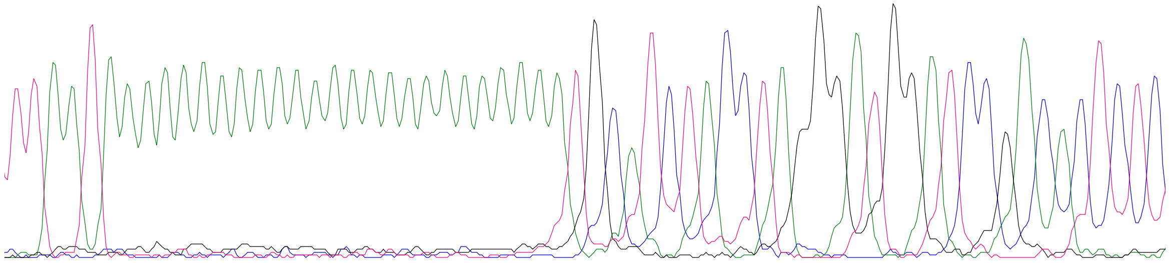

TTAATAAAAAAAAAAAAAAAAAAAAAAAAATGCATCTACCTAGGGATGGATCCGACACTCTC

Supplementary Data 2. FOXO1 expression vector sequence.


```
                                                10        20        30        40        50        60        70        80        90        100       110
                                                TTACTGGCTTATCGAAATTAATACGACTCACTATAGGGAGACCCAAGCTGGCTAGCGTTTAAACTTAAGCTTATGGCCGAGGCGCCTCAGGTGGTGGAGATCGACCCGGA
B07-W137766-C4901HF140-2-PEGFP-N-5.ab1(1>848) → TTACTGGCTTATCGAAATTAATACGACTCACTATAGGGAGACCCAAGCTGGCTAGcGtTTAAACTTAAGCTTATGGCCGAGGCGCCTCAGGTGGTGGAGATCGACCCGGA
C4901HF140-2.seq(1>1968)                      →                                                                         ATGGCCGAGGCGCCTCAGGTGGTGGAGATCGACCCGGA

                                                120       130       140       150       160       170       180       190       200       210       220
                                                CTTCGAGCCGCTGCCCCGGCCGCGCTCGTGCACCTGGCCGCTGCCCAGGCCGGAGTTTAGCCAGTCCAACTCGGCCACCTCCAGCCCGGCGCCGTCGGGCAGCGCGGCTG
B07-W137766-C4901HF140-2-PEGFP-N-5.ab1(1>848) → CTTCGAGCCGCTGCCCCGGCCGCGCTCGTGCACCTGGCCGCTGCCCAGGCCGGAGTTTAGCCAGTCCAACTCGGCCACCTCCAGCCCGGCGCCGTCGGGCAGCGCGGCTG
C4901HF140-2.seq(1>1968)                      → CTTCGAGCCGCTGCCCCGGCCGCGCTCGTGCACCTGGCCGCTGCCCAGGCCGGAGTTTAGCCAGTCCAACTCGGCCACCTCCAGCCCGGCGCCGTCGGGCAGCGCGGCTG

                                                230       240       250       260       270       280       290       300       310       320       330
                                                CCAACCCCGACGCCGCGGCGGGCCTGCCCTCGGCCTCGGCTGCCGCTGTCAGCGCCGACTTCATGAGCAACCTGAGCTTGCTGGAGGAGAGCGAGGACTTCCCGCAGGCG
B07-W137766-C4901HF140-2-PEGFP-N-5.ab1(1>848) → CCAACCCCGACGCCGCGGCGGGCCTGCCCTCGGCCTCGGCTGCCGCTGTCAGCGCCGACTTCATGAGCAACCTGAGCTTGCTGGAGGAGAGCGAGGACTTCCCGCAGGCG
C4901HF140-2.seq(1>1968)                      → CCAACCCCGACGCCGCGGCGGGCCTGCCCTCGGCCTCGGCTGCCGCTGTCAGCGCCGACTTCATGAGCAACCTGAGCTTGCTGGAGGAGAGCGAGGACTTCCCGCAGGCG

                                                340       350       360       370       380       390       400       410       420       430       440
                                                CCCGGCTCCGTGGCGGCGGCGGTGGCGGCGGCGGCCGCCGCGGCCGCCACCGGGGGGCTGTGCGGGGACTTCCAGGGCCCGGAGGCGGGCTGCCTGCACCCAGCGCCACC
B07-W137766-C4901HF140-2-PEGFP-N-5.ab1(1>848) → CCCGGCTCCGTGGCGGCGGCGGTGGCGGCGGCGGCCGCCGCGGCCGCCACCGGGGGGCTGTGCGGGGACTTCCAGGGCCCGGAGGCGGGCTGCCTGCACCCAGCGCCACC
C4901HF140-2.seq(1>1968)                      → CCCGGCTCCGTGGCGGCGGCGGTGGCGGCGGCGGCCGCCGCGGCCGCCACCGGGGGGCTGTGCGGGGACTTCCAGGGCCCGGAGGCGGGCTGCCTGCACCCAGCGCCACC

                                                450       460       470       480       490       500       510       520       530       540       550
                                                GCAGCCCCCGCCGCCCGGGCCGCTGTCGCAGCACCCGCCGGTGCCCCCCGCCGCCGCTGGGCCGCTCGCGGGGCAGCCGCGCAAGAGCAGCTCGTCCCGCCGCAACGCGT
B07-W137766-C4901HF140-2-PEGFP-N-5.ab1(1>848) → GCAGCCCCCGCCGCCCGGGCCGCTGTCGCAGCACCCGCCGGTGCCCCCCGCCGCCGCTGGGCCGCTCGCGGGGCAGCCGCGCAAGAGCAGCTCGTCCCGCCGCAACGCGT
C4901HF140-2.seq(1>1968)                      → GCAGCCCCCGCCGCCCGGGCCGCTGTCGCAGCACCCGCCGGTGCCCCCCGCCGCCGCTGGGCCGCTCGCGGGGCAGCCGCGCAAGAGCAGCTCGTCCCGCCGCAACGCGT

                                                560       570       580       590       600       610       620       630       640       650       660
                                                GGGGCAACCTGTCCTACGCCGACCTCATCACCAAGGCCATCGAGAGCTCGGCGGAGAAGCGGCTCACGCTGTCGCAGATCTACGAGTGGATGGTCAAGAGCGTGCCCTAC
B07-W137766-C4901HF140-2-PEGFP-N-5.ab1(1>848) → GGGGCAACCTGTCCTACGCCGACCTCATCACCAAGGCCATCGAGAGCTCGGCGGAGAAGCGGCTCACGCTGTCGCAGATCTACGAGTGGATGGTCAAGAGCGTGCCCTAC
C4901HF140-2.seq(1>1968)                      → GGGGCAACCTGTCCTACGCCGACCTCATCACCAAGGCCATCGAGAGCTCGGCGGAGAAGCGGCTCACGCTGTCGCAGATCTACGAGTGGATGGTCAAGAGCGTGCCCTAC

                                                670       680       690       700       710       720       730       740       750       760       770
                                                TTCAAGGATAAGGGTGACAGCAACAGCTCGGCGGGCTGGAAGAATTCAATTCGTCATAATCTGTCCCTACACAGCAAGTTCATTCGTGTGCAGAATGAAGGAACTGGAAA
B07-W137766-C4901HF140-2-PEGFP-N-5.ab1(1>848) → TTCAAGGATAAGGGTGACAGCAACAGCTCGGCGGGCTGGAAGAATTCAATTCGTCATAATCTGTCCCTACACAGCAAGTTCATTCGTGTGCAGAATGAAGGAACTGGAAA
C4901HF140-2.seq(1>1968)                      → TTCAAGGATAAGGGTGACAGCAACAGCTCGGCGGGCTGGAAGAATTCAATTCGTCATAATCTGTCCCTACACAGCAAGTTCATTCGTGTGCAGAATGAAGGAACTGGAAA
E06-W137766-C4901HF140-2-2-SEQ1F.ab1(1>760)   →                                                             CTGTCCCTACACAGCAAGTTCATTCGTGTGCAGAATGAAGGAACTGGAAA

                                                780       790       800       810       820       830       840       850       860       870       880
                                                AAGTTCTTGGTGGATGCTCAATCCAGAGGGTGGCAAGAGCGGGAAATCTCCTAGGAGAAGAGCTGCATCCATGGACAACAACAGTAAATTTGCTAAGAGCCGAAGCCGAG
B07-W137766-C4901HF140-2-PEGFP-N-5.ab1(1>848) → AAGTTCTTGGTGGATGCTCAATCCAGAGGGTGGCAAGAGCGGGAAATCTCCTAGGAGAaGAGCTGCATCCATGGACaA
C4901HF140-2.seq(1>1968)                      → AAGTTCTTGGTGGATGCTCAATCCAGAGGGTGGCAAGAGCGGGAAATCTCCTAGGAGAAGAGCTGCATCCATGGACAACAACAGTAAATTTGCTAAGAGCCGAAGCCGAG
E06-W137766-C4901HF140-2-2-SEQ1F.ab1(1>760)   → AAGTTCTTGGTGGATGCTCAATCCAGAGGGTGGCAAGAGCGGGAAATCTCCTAGGAGAAGAGCTGCATCCATGGACAACAACAGTAAATTTGCTAAGAGCCGAAGCCGAG
```

```
                                                    890       900       910       920       930       940       950       960       970       980       990
                                                    CTGCCAAGAAGAAAGCATCTCTCCAGTCTGGCCAGGAGGGTGCTGGGGACAGCCCTGGATCACAGTTTTCCAAATGGCCTGCAAGCCCTGGCTCTCACAGCAATGATGAC
C4901HF140-2.seq(1>1968)                        →   CTGCCAAGAAGAAAGCATCTCTCCAGTCTGGCCAGGAGGGTGCTGGGGACAGCCCTGGATCACAGTTTTCCAAATGGCCTGCAAGCCCTGGCTCTCACAGCAATGATGAC
E06-W137766-C4901HF140-2-2-SEQ1F.ab1(1>760)     →   CTGCCAAGAAGAAAGCATCTCTCCAGTCTGGCCAGGAGGGTGCTGGGGACAGCCCTGGATCACAGTTTTCCAAATGGCCTGCAAGCCCTGGCTCTCACAGCAATGATGAC

                                                   1000      1010      1020      1030      1040      1050      1060      1070      1080      1090      1100
                                                    TTTGATAACTGGAGTACATTTCGCCCTCGAACTAGCTCAAATGCTAGTACTATTAGTGGGAGACTCTCACCCATTATGACCGAACAGGATGATCTTGGAGAAGGGGATGT
C4901HF140-2.seq(1>1968)                        →   TTTGATAACTGGAGTACATTTCGCCCTCGAACTAGCTCAAATGCTAGTACTATTAGTGGGAGACTCTCACCCATTATGACCGAACAGGATGATCTTGGAGAAGGGGATGT
E06-W137766-C4901HF140-2-2-SEQ1F.ab1(1>760)     →   TTTGATAACTGGAGTACATTTCGCCCTCGAACTAGCTCAAATGCTAGTACTATTAGTGGGAGACTCTCACCCATTATGACCGAACAGGATGATCTTGGAGAAGGGGATGT

                                                   1110      1120      1130      1140      1150      1160      1170      1180      1190      1200      1210
                                                    GCATTCTATGGTGTACCCGCCATCTGCCGCAAAGATGGCCTCTACTTTACCCAGTCTGTCTGAGATAAGCAATCCCGAAAACATGGAAAATCTTTTGGATAATCTCAACC
C4901HF140-2.seq(1>1968)                        →   GCATTCTATGGTGTACCCGCCATCTGCCGCAAAGATGGCCTCTACTTTACCCAGTCTGTCTGAGATAAGCAATCCCGAAAACATGGAAAATCTTTTGGATAATCTCAACC
E06-W137766-C4901HF140-2-2-SEQ1F.ab1(1>760)     →   GCATTCTATGGTGTACCCGCCATCTGCCGCAAAGATGGCCTCTACTTTACCCAGTCTGTCTGAGATAAGCAATCCCGAAAACATGGAAAATCTTTTGGATAATCTCAACC

                                                   1220      1230      1240      1250      1260      1270      1280      1290      1300      1310      1320
                                                    TTCTCTCATCACCAACATCATTAACTGTTTCGACCCAGTCCTCACCTGGCACCATGATGCAGCAGACGCCGTGCTACTCGTTTGCGCCACCAAACACCAGTTTGAATTCA
C4901HF140-2.seq(1>1968)                        →   TTCTCTCATCACCAACATCATTAACTGTTTCGACCCAGTCCTCACCTGGCACCATGATGCAGCAGACGCCGTGCTACTCGTTTGCGCCACCAAACACCAGTTTGAATTCA
E06-W137766-C4901HF140-2-2-SEQ1F.ab1(1>760)     →   TTCTCTCATCACCAACATCATTAACTGTTTCGACCCAGTCCTCACCTGGCACCATGATGCAGCAGACGCCGTGCTACTCGTTTGCGCCACCAAACACCAGTTTGAATTCA

                                                   1330      1340      1350      1360      1370      1380      1390      1400      1410      1420      1430
                                                    CCCAGCCCAAACTACCAAAAATATACATATGGCCAATCCAGCATGAGCCCTTTGCCCCAGATGCCTATACAAACACTTCAGGACAATAAGTCGAGTTATGGAGGTATGAG
C4901HF140-2.seq(1>1968)                        →   CCCAGCCCAAACTACCAAAAATATACATATGGCCAATCCAGCATGAGCCCTTTGCCCCAGATGCCTATACAAACACTTCAGGACAATAAGTCGAGTTATGGAGGTATGAG
E06-W137766-C4901HF140-2-2-SEQ1F.ab1(1>760)     →   CCCAGCCCAAACTACCAAAAATATACATATGGCCAATCCAGCATGAGCCCTTTGCCCCAGATGCCTATACAAACACTTCAGGACAATAAGTCGAGTTATGGAGGTATGAG

                                                   1440      1450      1460      1470      1480      1490      1500      1510      1520      1530      1540
                                                    TCAGTATAACTGTGCGCCTGGACTCTTGAAGGAGTTGCTGACTTCTGACTCTCCTCCCCATAATGACATTATGACACCAGTTGATCCTGGGGTAGCCCAGCCCAACAGCC
C4901HF140-2.seq(1>1968)                        →   TCAGTATAACTGTGCGCCTGGACTCTTGAAGGAGTTGCTGACTTCTGACTCTCCTCCCCATAATGACATTATGACACCAGTTGATCCTGGGGTAGCCCAGCCCAACAGCC
E06-W137766-C4901HF140-2-2-SEQ1F.ab1(1>760)     →   TCAGTATAACTGTGCGCCTGGACTCTTGAAGGAGTTGCTGACTTCTGACT
E07-W137766-C4901HF140-2-2-SEQ2F.ab1(1>766)     →                                AGGAGTTGCTGACTTCTGACTCTCCTCCCCATAATGACATTATGACACCAGTTGATCCTGGGGTAGCCCAGCCCAACAGCC

                                                   1550      1560      1570      1580      1590      1600      1610      1620      1630      1640      1650
                                                    GGGTTCTGGGCCAGAACGTCATGATGGGCCCTAATTCGGTCATGTCAACCTATGGCAGCCAGGCATCTCATAACAAAATGATGAATCCCAGCTCCCATACCCACCCTGGA
C4901HF140-2.seq(1>1968)                        →   GGGTTCTGGGCCAGAACGTCATGATGGGCCCTAATTCGGTCATGTCAACCTATGGCAGCCAGGCATCTCATAACAAAATGATGAATCCCAGCTCCCATACCCACCCTGGA
E07-W137766-C4901HF140-2-2-SEQ2F.ab1(1>766)     →   GGGTTCTGGGCCAGAACGTCATGATGGGCCCTAATTCGGTCATGTCAACCTATGGCAGCCAGGCATCTCATAACAAAATGATGAATCCCAGCTCCCATACCCACCCTGGA

                                                   1660      1670      1680      1690      1700      1710      1720      1730      1740      1750      1760
                                                    CATGCTCAGCAGACATCTGCAGTTAACGGGCGTCCCCTGCCCCACACGGTAAGCACCATGCCCCACACCTCGGGTATGAACCGCCTGACCCAAGTGAAGACACCTGTACA
C4901HF140-2.seq(1>1968)                        →   CATGCTCAGCAGACATCTGCAGTTAACGGGCGTCCCCTGCCCCACACGGTAAGCACCATGCCCCACACCTCGGGTATGAACCGCCTGACCCAAGTGAAGACACCTGTACA
E07-W137766-C4901HF140-2-2-SEQ2F.ab1(1>766)     →   CATGCTCAGCAGACATCTGCAGTTAACGGGCGTCCCCTGCCCCACACGGTAAGCACCATGCCCCACACCTCGGGTATGAACCGCCTGACCCAAGTGAAGACACCTGTACA
```

```
                                              1770      1780      1790      1800      1810      1820      1830      1840      1850      1860      1870
                                         AGTGCCTCTGCCCCACCCCATGCAGATGAGTGCCCTGGGGGGCTACTCCTCCGTGAGCAGCTGCAATGGCTATGGCAGAATGGGCCTTCTCCACCAGGAGAAGCTCCCAA
C4901HF140-2.seq(1>1968)              →  AGTGCCTCTGCCCCACCCCATGCAGATGAGTGCCCTGGGGGGCTACTCCTCCGTGAGCAGCTGCAATGGCTATGGCAGAATGGGCCTTCTCCACCAGGAGAAGCTCCCAA
E07-W137766-C4901HF140-2-2-SEQ2F.ab1(1>766) →  AGTGCCTCTGCCCCACCCCATGCAGATGAGTGCCCTGGGGGGCTACTCCTCCGTGAGCAGCTGCAATGGCTATGGCAGAATGGGCCTTCTCCACCAGGAGAAGCTCCCAA

                                              1880      1890      1900      1910      1920      1930      1940      1950      1960      1970      1980
                                         GTGACTTGGATGGCATGTTCATTGAGCGCTTAGACTGTGACATGGAATCCATCATTCGGAATGACCTCATGGATGGAGATACATTGGATTTTAACTTTGACAATGTGTTG
C4901HF140-2.seq(1>1968)              →  GTGACTTGGATGGCATGTTCATTGAGCGCTTAGACTGTGACATGGAATCCATCATTCGGAATGACCTCATGGATGGAGATACATTGGATTTTAACTTTGACAATGTGTTG
E07-W137766-C4901HF140-2-2-SEQ2F.ab1(1>766) →  GTGACTTGGATGGCATGTTCATTGAGCGCTTAGACTGTGACATGGAATCCATCATTCGGAATGACCTCATGGATGGAGATACATTGGATTTTAACTTTGACAATGTGTTG

                                              1990      2000      2010      2020      2030      2040      2050      2060      2070      2080      2090
                                         CCCAACCAAAGCTTCCCACACAGTGTCAAGACAACGACACATAGCTGGGTGTCAGGCTGAGGTACCGAGCTCGGATCCACTAGTCCAGTGTGGTGGAATTCTGCAGATAT
C4901HF140-2.seq(1>1968)              →  CCCAACCAAAGCTTCCCACACAGTGTCAAGACAACGACACATAGCTGGGTGTCAGGCTGA
E07-W137766-C4901HF140-2-2-SEQ2F.ab1(1>766) →  CCCAACCAAAGCTTCCCACACAGTGTCAAGACAACGACACATAGCTGGGTGTCAGGCTGAGGTACCGAGCTCGGATCCACTAGTCCAGTGTGGTGGAATTCTGCAGATAT

                                              2100      2110      2120      2130      2140      2150      2160      2170      2180      2190      2200
                                         CCAGCACAGTGGCGGCCGCTCGAGTCTAGAGGGCCCGTTTAAACCCGCTGATCAGCCTCGACTGTGCCTTCTAGTTGCCAGCCATCTGTTGTTTGCCCCTCCCCCGTGCC
E07-W137766-C4901HF140-2-2-SEQ2F.ab1(1>766) →  CCAGCACAGTGGCGGCCGCTCGAGTCTAGAGGGCCCGTTTAAACCCGCTGATCAGCCTCGACTGTGCCTTCTAGTTGCCAGCCATCTGTTGTTTGCCCCTCCCCCGTGCC

                                              2210      2220
                                         TTCCTTGACCCTGGAAGGTGCCACT
E07-W137766-C4901HF140-2-2-SEQ2F.ab1(1>766) →  TTCCTTGACCCTGGAAGGTGCCACT
```

Supplementary Fig.1 Expression characteristics of CYP1B1-AS1.

a

Protein coding potential

LNCipedia version 5.2

Metric	Raw result	Interpretation
PRIDE reprocessing 2.0	0	non-coding
Lee translation initiation sites	0	non-coding
PhyloCSF score	-77.3697	non-coding
CPAT coding probability	16.57%	non-coding
Bazzini small ORFs	0	non-coding

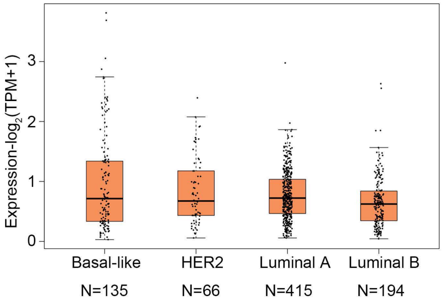

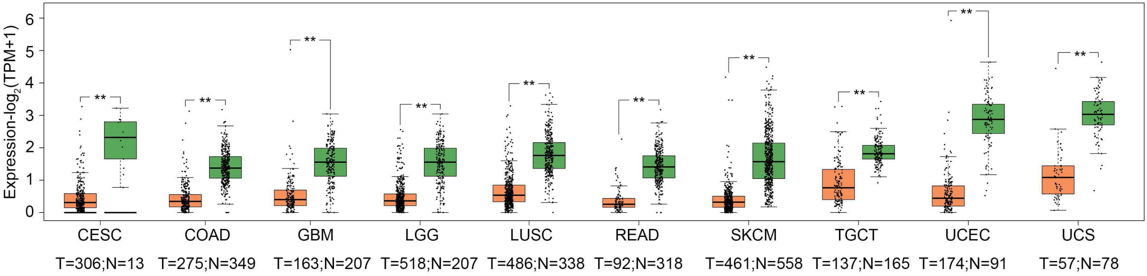

(a) The LNCipedia database shows the coding capacity and conserved features of CYP1B1-AS1. (b) TCGA data were used to analyze the expression of CYP1B1-AS1 across molecular subtypes of breast cancer. (c) TCGA data show that CYP1B1-AS1 is downregulated in various cancers. CESC, cervical and endocervical cancers. COAD, colon adenocarcinoma. GBM, Glioblastoma multiforme. LGG, brain lower grade glioma. LUSC, lung squamous cell carcinoma. READ, rectum adenocarcinoma. SKCM, skin cutaneous melanoma. TGTC, testicular germ cell tumor. UCEC, uterine corpus endometrial carcinoma. UCS, uterine carcinosarcoma. ***P* < 0.01.

Supplementary Fig. 2 Analysis of CYP1B1-AS1 expression regulation.

a

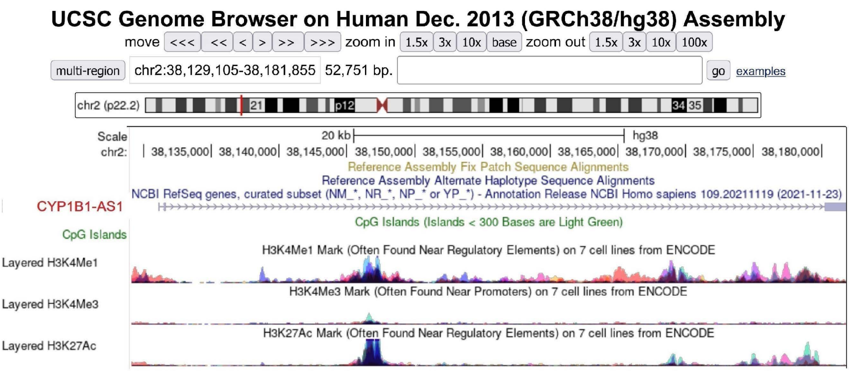

b

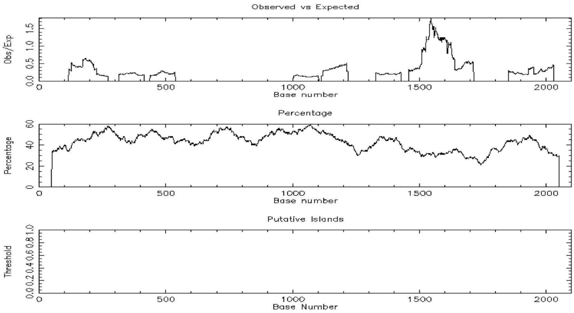

c

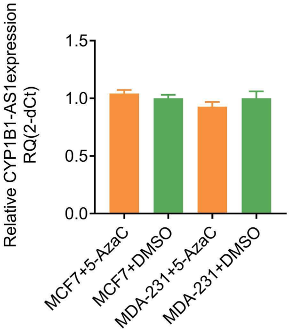

d

JASPAR2022

Matrix ID	Name	Score	Relative score	Sequence ID	Start	End	Strand	Predicted sequence
MA1947.1	MA1947.1.ETV5::FOXO1	11.932848	0.8925321454612196	NC_000002.12:38129105-38181855	1881	1893	+	AACAGCAGGAAGC
MA1936.1	MA1936.1.ERF::FOXO1	11.498526	0.8851013664643971	NC_000002.12:38129105-38181855	1881	1894	+	AACAGCAGGAAGCA
MA1936.1	MA1936.1.ERF::FOXO1	7.133964	0.8202557469227999	NC_000002.12:38129105-38181855	1208	1221	+	ATAAGCAGAAAAAT
MA1947.1	MA1947.1.ETV5::FOXO1	7.5240784	0.8158957888793826	NC_000002.12:38129105-38181855	744	756	+	GTTCACAAGATGT
MA1936.1	MA1936.1.ERF::FOXO1	6.1716366	0.8059581546361436	NC_000002.12:38129105-38181855	1479	1492	+	TTATACAGTAAGTT
MA1947.1	MA1947.1.ETV5::FOXO1	6.630751	0.8003673420087025	NC_000002.12:38129105-38181855	1868	1880	+	ATCAACATGAGAC

(a) Characterization of CYP1B1-AS1 gene and its promoter region provided by UCSC Genome Browser. (b) Methylation of the CYP1B1-AS1 promoter region predicted by EBI CpGplot. (c) qPCR was used to detect changes in CYP1B1-AS1 after treatment of MCF7 and MDA-MB-231 cells with the demethylating drug 5-Azacytidine. 5-Azac, 5-Azacytidine. Solvent DMSO as blank control. Experiments were performed at least 3 times. Data are presented as mean ± SEM and analyzed with a two-tailed *T* test. (d) JASPAR was used to analyze the binding sites of the transcription factor FOXO1 to the CYP1B1-AS1 promoter region.

Supplementary Fig.3 The results of the co-immunoprecipitation assay.

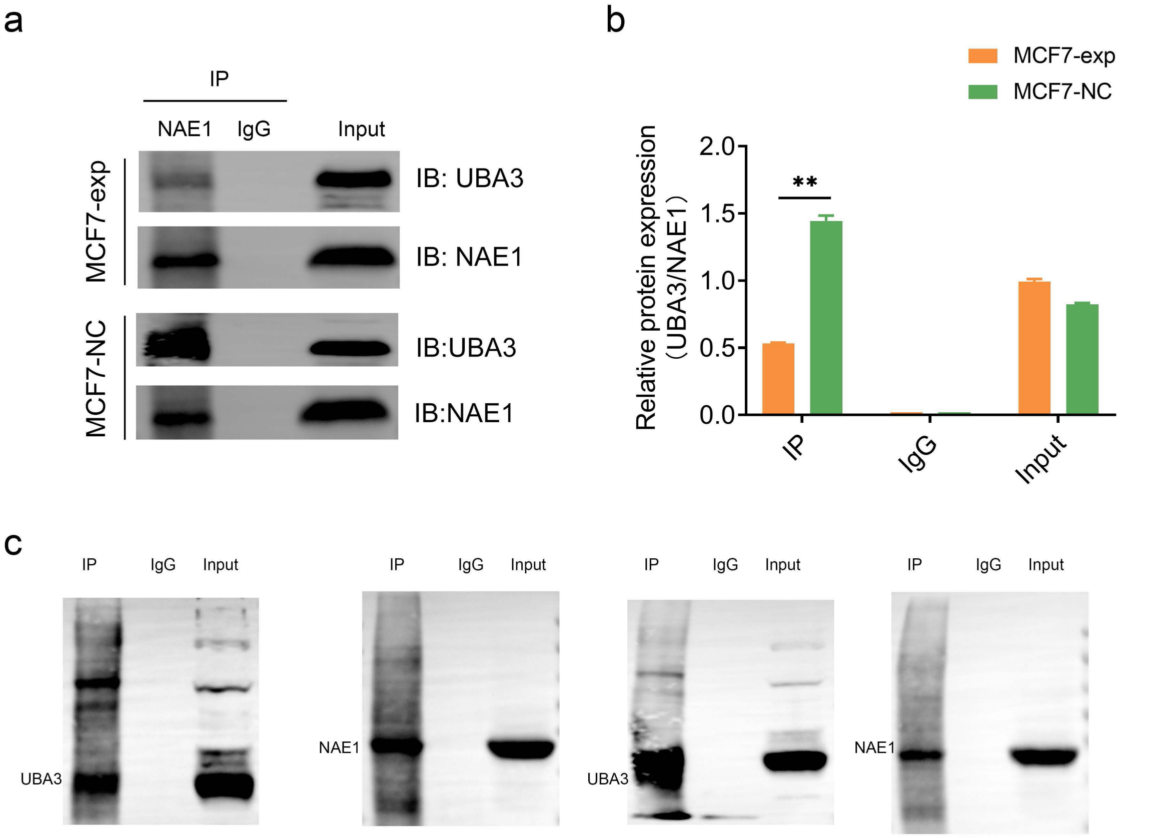

(a,b) Co-immunoprecipitation experiments were used to analyze changes in the amount of UBA3 protein bound to NAE1 in MCF7 cells after CYP1B1-AS1 upregulation. **$P < 0.01$. (c) Raw Western blot data from co-immunoprecipitation experiments.

Supplementary Fig.4 Raw data of Western blot.

Fig.2f

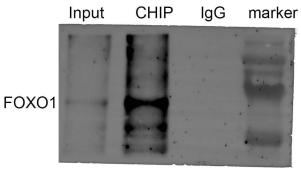

Fig.2i

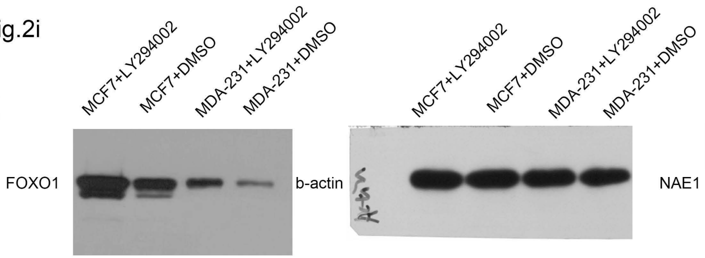

Fig.5e

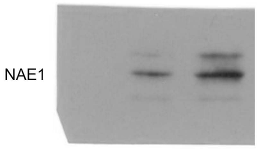

Fig.6a

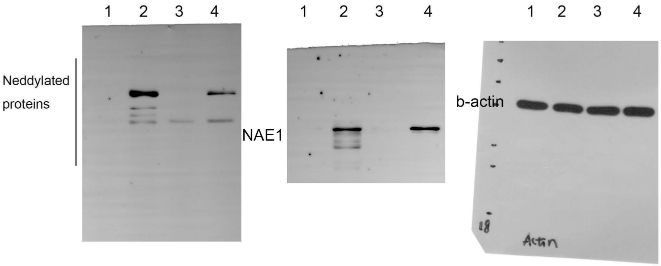

Fig.6c

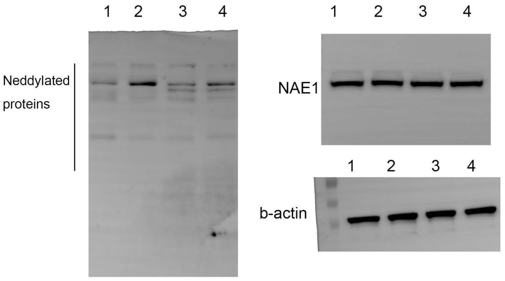

Fig.6g

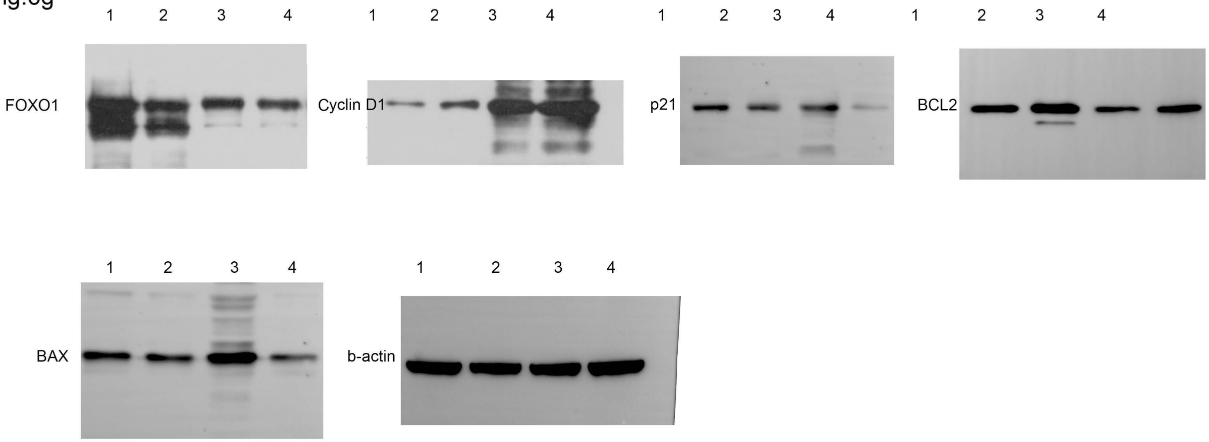

1, MCF7-exp; 2, MCF7-NC; 3, MDA-231-exp; 4, MDA-231-NC.
